# Supplementary material for: NBEAL2 deficiency in humans leads to low CTLA-4 expression in activated conventional T cells
Source: Nat Commun. 2023 Jun 22;14:3728. doi: 10.1038/s41467-023-39295-7 (PMC10287742; doi:10.1038/s41467-023-39295-7)
Supplement: Supplementary file 1 — Supplementary Information [file 41467_2023_39295_MOESM1_ESM.pdf]

Supplementary information: NBEAL2 deficiency in humans leads to low CTLA-4 expression in activated conventional T cells

Supplementary Information

This PDF includes:

Supplementary Figures S1 to S12.

Supplementary Tables S1 to S9.

Supplementary methods.

Supplementary figures:

Supplementary Figure 1. **Family trees of the cohort and Vitamin B12 and FasL assays**

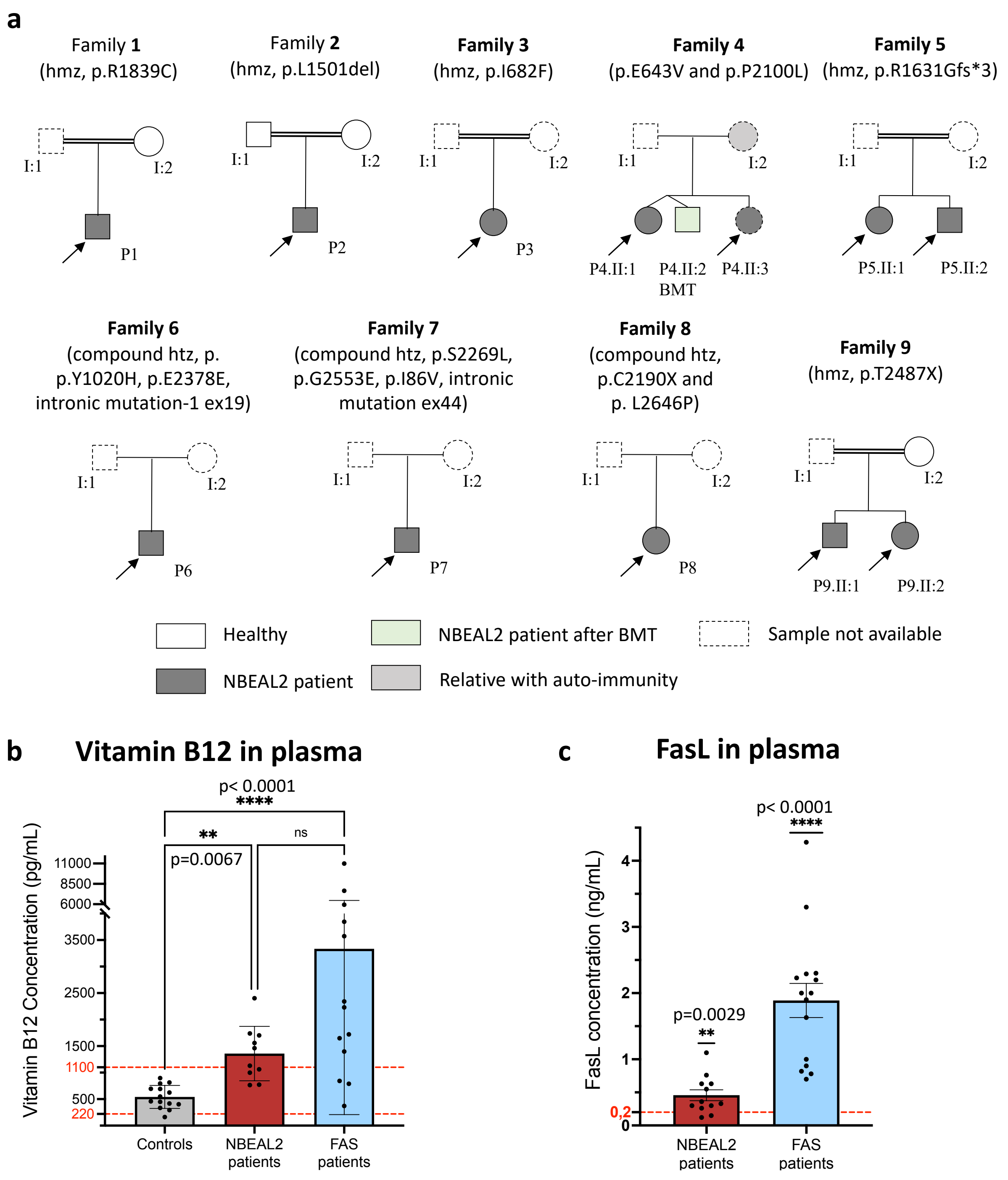

Supplementary Figure 2. CyTOF analyses – clustering and immune phenotype of GPS patients.

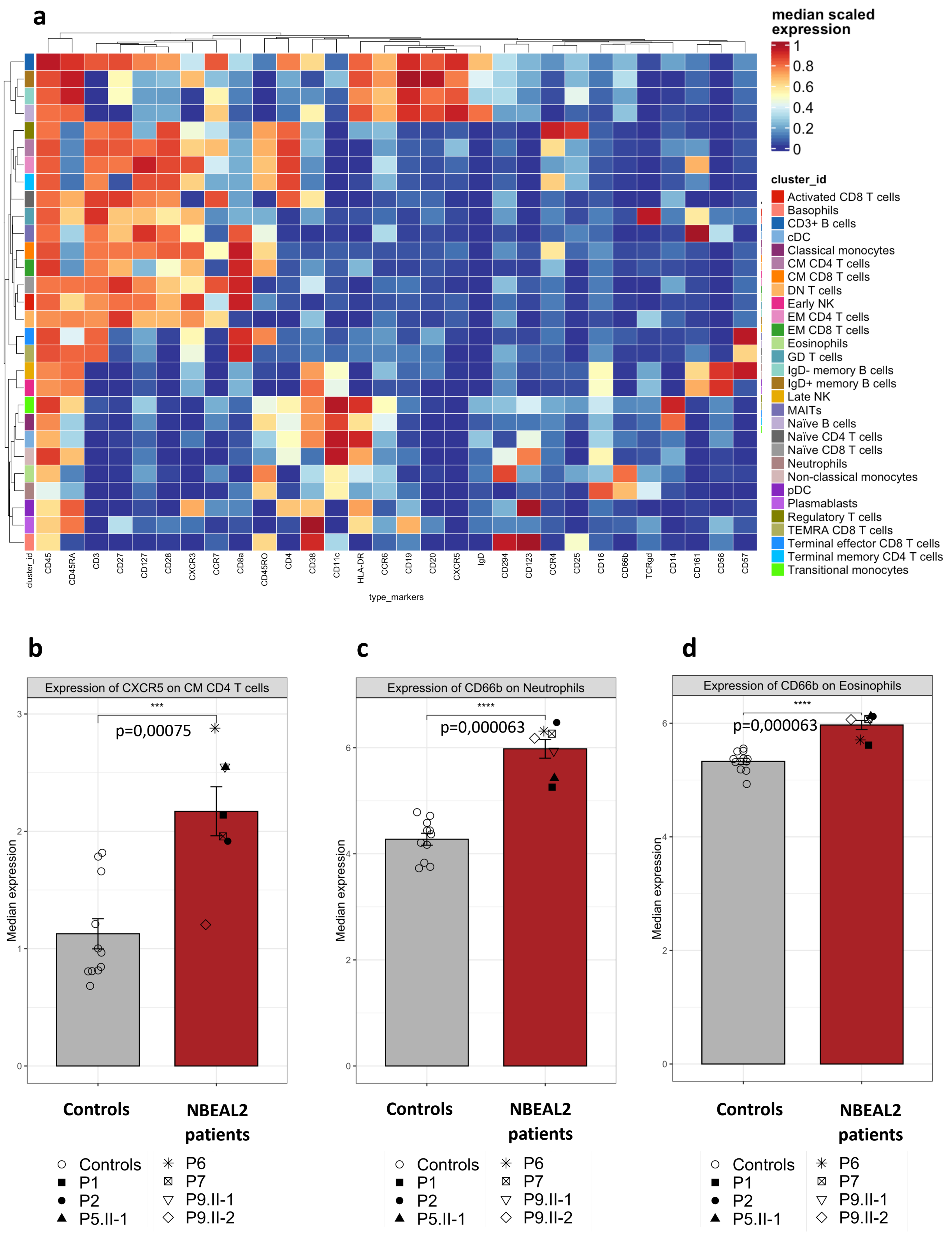

Supplementary Figure 2. **CyTOF analyses – clustering and immune phenotype of GPS patients. (a)** Heatmap of intensities for each lineage marker used for cluster identification after unsupervised clustering for all samples. **(b)** Expression of CXCR5 in central memory (CM) CD4+ T cells from controls (N=11) and GPS patients (N= 7, NBEAL2 group). Each dot corresponds to a donor. Mean +/-SD is represented. These data were obtained after CyTOF experiment, performed once for each sample. Two-tailed p-values were determined with a nonparametric Mann-whitney test. (*) p-value < 0.05; (**) p-value < 0.01 ; (***) p-value < 0.005 ; (****) p-value < 0.0001. **(c)** Expression of CD66b on neutrophils from controls (N=11) and GPS patients (N=7, NBEAL2 group). Each dot corresponds to a donor. Mean +/-SD is represented. These data were obtained after CyTOF experiment, performed once for each sample. Two-tailed p-values were determined with a nonparametric Mann-whitney test. (*) p-value < 0.05; (**) p-value < 0.01 ; (***) p-value < 0.005 ; (****) p-value < 0.0001. **(d)** Expression of CD66b on eosinophils from controls (N=11) and GPS patients (N=7, NBEAL2 group. Each dot corresponds to a donor. Mean +/-SD is represented. These data were obtained after CyTOF experiment, performed once for each sample. Two-tailed p-values were determined with a nonparametric Mann-whitney test. (*) p-value < 0.05; (**) p-value < 0.01 ; (***) p-value < 0.005 ; (****) p-value < 0.0001.

Supplementary Figure 3. Mass spectrometry analyses – strategy of proteins selection.

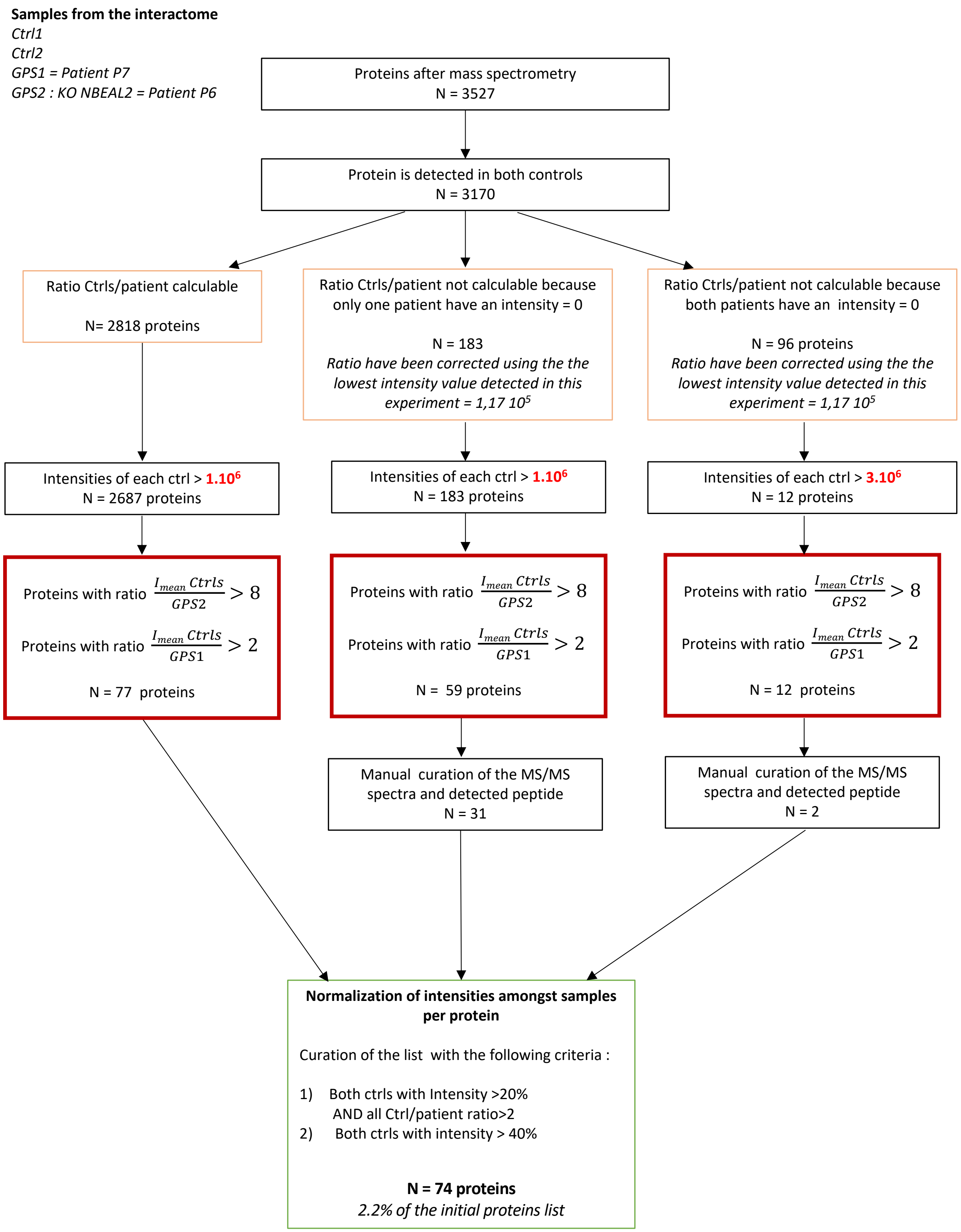

Supplementary Figure 3. Mass spectrometry analyses – strategy of proteins selection. Strategy of selection of the 74 partners of NBEAL2 obtained from the interactome by mass spectrometry analysis.

41 **Supplementary Figure 4. Treg staining**

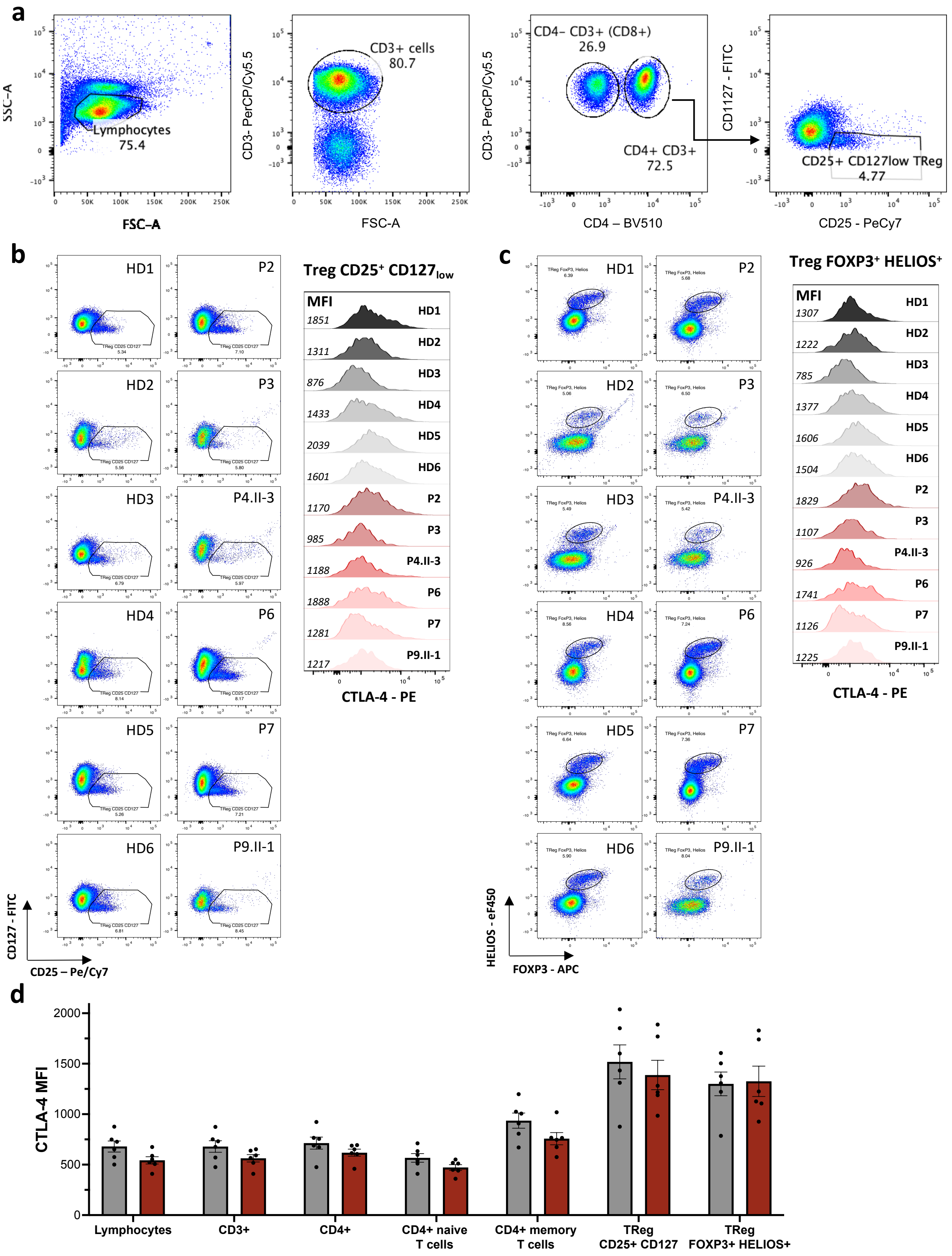

42

Supplementary Figure 4. Tregs staining. (a) Gating strategy to identify Tregs in unstimulated PBMC. After gating on lymphocytes, $CD3^+$ T cells, then $CD4^+$ T cells were selected. Tregs are defined among $CD4^+$ T cells by $CD25^+$ $CD127_{low}$ staining. **(b)** Dot plots of CD4+ Tregs defined by $CD25^+$ $CD127_{low}$ staining, for tested patients and controls. Histograms of CTLA-4 staining and mean of fluorescence intensity (MFI) value of CTLA-4 per sample. **(c)** Dot plots of CD4+ Tregs defined by $FOXP3^+$ $HELIOS^+$ staining, for tested patients and controls. Histograms of CTLA-4 staining and mean of fluorescence intensity (MFI) value of CTLA-4 per sample. **(d)** Mean of fluorescence intensity (MFI) values of CTLA-4 for lymphocytes, CD3+ cells, CD4+ T cells, naïve CD45RA+ CD4+ T cells, memory CD45RA- CD4+ T cells, CD25+ CD127low Treg and FOXP3+ HELIOS+ Treg for controls (N=6) and NBEAL2 patients (N=6). Each dot corresponds to an idependant sample. Staining experiment was performed once. Mean +/-SEM is represented.

Supplementary Figure 5: activated T cells - gating strategy

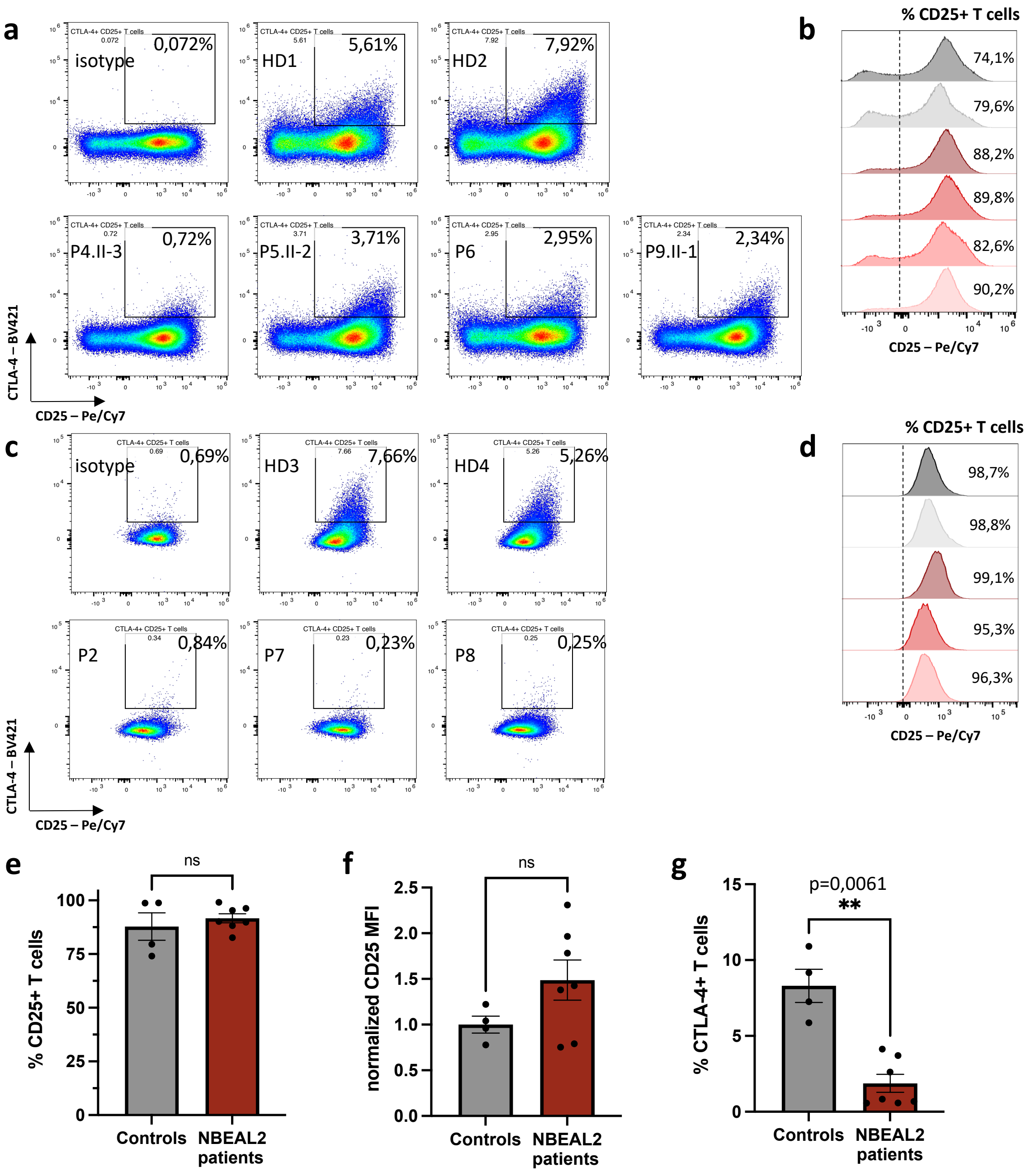

Supplementary Figure 5: activated T cells - gating strategy. (a) Gating strategy to define CTLA-4 CD25 positive T cells after activation. The gating was tuned using a control isotype antibody. Dot plots of CTLA-4 versus CD25 of activated T cells, for tested patients and controls in a first experiment are shown. Percentages are indicated on the top. **(b)** Histograms of CD25 staining and percentage of $CD25^{+}$ activated T cells per sample. **(c)** Gating strategy to define CTLA-4 CD25 positive T cells after activation. The gating was tuned using a control isotype antibody. Dot plots of CTLA-4 versus CD25 of activated T cells, for tested patients and controls in a second experiment are shown. Percentages are indicated on the top. **(d)** Histograms of CD25

staining and percentage of $CD25^+$ activated T cells per sample. **(e)** Percentage of $CD25^+$ activated T cells of controls (N=4) or NBEAL2 patients (N=7) activated T cells. Mean +/-SEM is represented. According to a two-tailed unpaired nonparametric Mann-Whitney test, there is no significant (ns) difference between the two groups. **(f)** Mean of fluorescence intensity (MFI) of CD25 in activated T cells of controls (N=4) or NBEAL2 patients (N=7). Mean +/-SEM is represented. According to a two-tailed unpaired nonparametric Mann-Whitney test, there is no significant (ns) difference between the two groups. (**g)** Percentage of activated T cells expressing CTLA-4 following TCR activation for controls (N=4) or NBEAL2 patients (N=7). Mean +/-SEM is represented. Two-tailed p-value is determined with a nonparametric Mann-Whitney test. (*) p-value < 0.05; (**) p-value < 0.01 ; (***) p-value < 0.005 ; (****) p-value < 0.0001.

Supplementary Figure 6: NBEAL2 and LRBA expression in sorted Tregs and conventional T cells from healthy donors

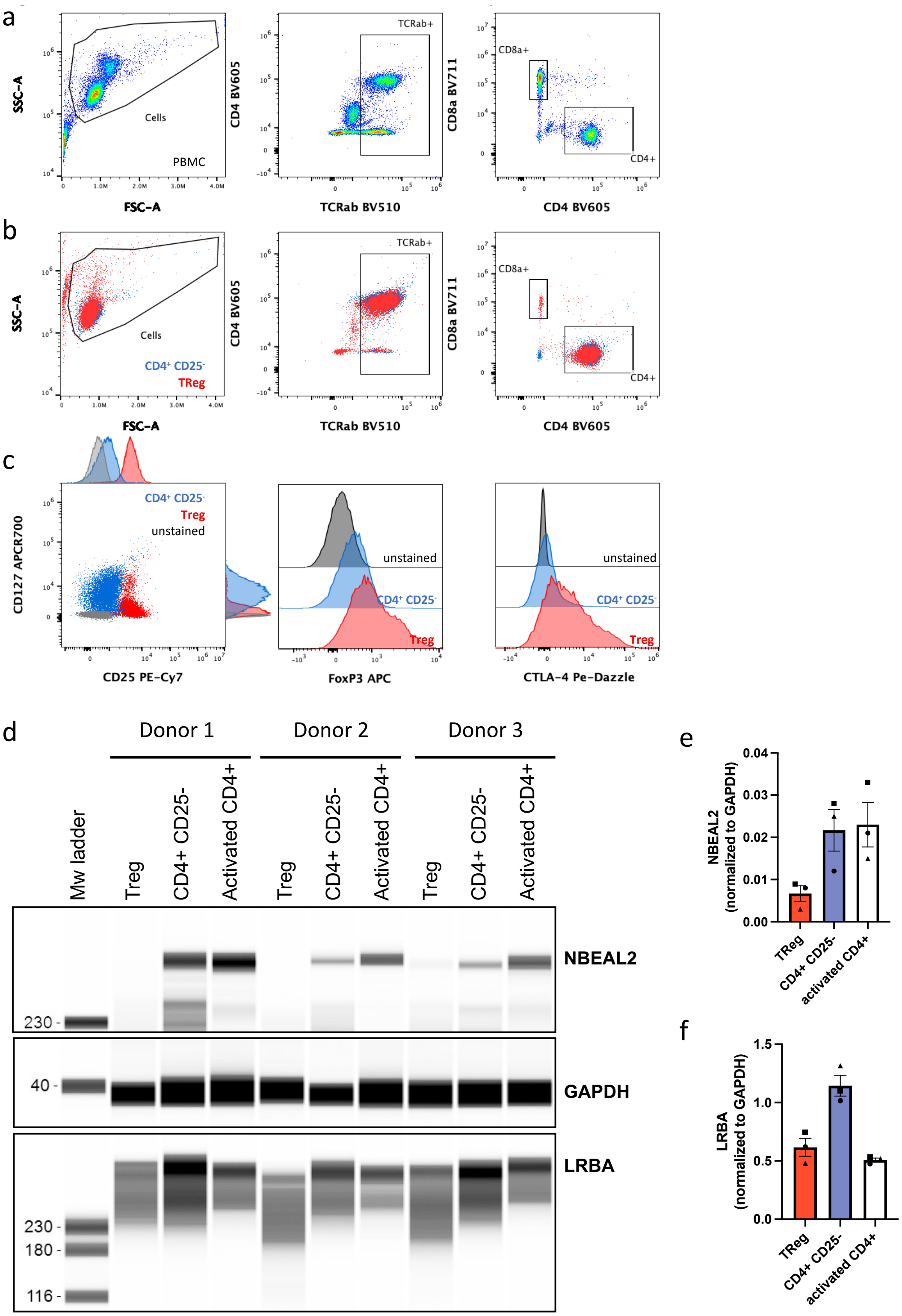

Supplementary Figure 6: NBEAL2 and LRBA expression in sorted Tregs and conventional T cells from healthy donors. (a) Example of the gating strategy of initial PBMC from a healthy

donor. **(b)** Gating strategy after sorting of Treg ($CD4^+$ $CD25^+$ CD127low) in red and $CD25^-$ $CD24^+$ T cells in blue. **(c)** Gating strategy and histograms illustrating the expression of the sorted cells, respectively Treg ($CD4^+$ $CD25^+$ CD127low) in red and $CD25^-$ $CD24^+$ T cells in blue. **(d)** Immunoblotting of NBEAL2, LRBA and GAPDH in different cell subtypes lysates from 3 healthy donors. Cell lysates are from either Treg, or non-activated $CD4^+$ $CD25^-$ T cells or $CD4^+$ T cells after TCR activation. Source data are provided as a Source Data file. **(e)** NBEAL2 expression normalized with GAPDH in Treg, non-activated $CD4^+$ $CD25^-$ T cells or $CD4^+$ T cells after TCR activation for three independent healthy donors. Means +/-SEM are represented. **(f)** LRBA expression normalized with GAPDH in Treg, non-activated $CD4^+$ $CD25^-$ T cells or $CD4^+$ T cells after TCR activation for three independent healthy donors. Means +/-SEM are represented.

Supplementary Figure 7. Immunoblotting of NBEAL2, LRBA and GAPDH – uncropped blots.

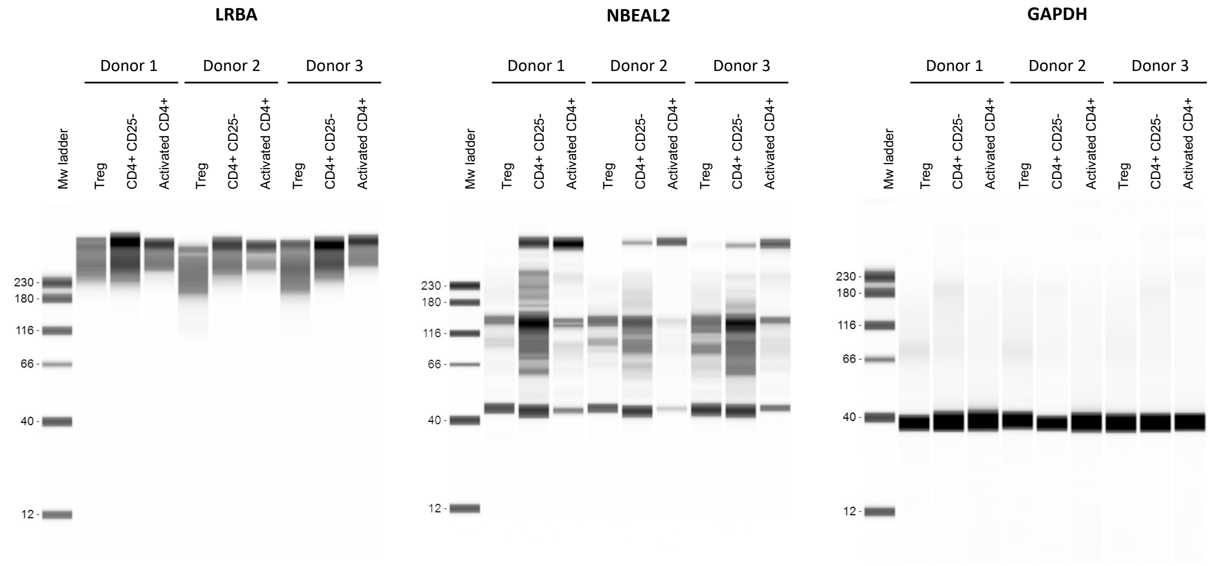

Supplementary Figure 7. Immunoblotting of NBEAL2, LRBA and GAPDH – uncropped blots. Source data of the immunoblotting of supplementary figure 6d. NBEAL2, LRBA and GAPDH in different cell subtypes lysates from 3 healthy donors. Cell lysates are from either Treg, or non-activated $CD4^{+}$ $CD25^{-}$ T cells or $CD4^{+}$ T cells after TCR activation.

Supplementary Figure 8: Single-cell RNA sequencing of NBEAL2- and LRBA-deficient cells

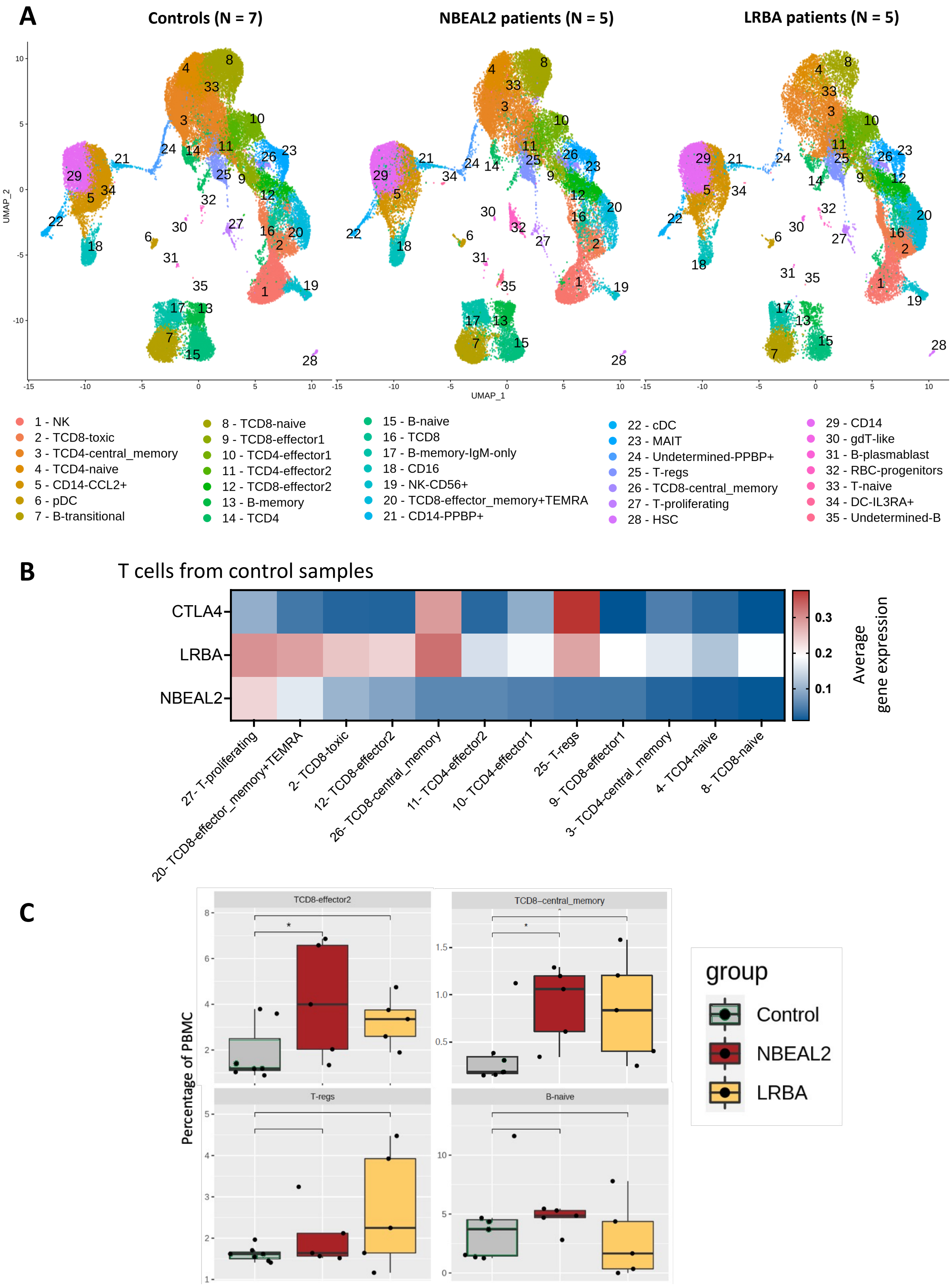

Supplementary Figure 8. Single-cell RNA sequencing of NBEAL2 and LRBA deficient cells. **(A)** PBMC from controls, NBEAL2 and LRBA patients were analyzed by single cell RNA Sequencing (scRNA Seq). UniformManifold Approximation and Projection (UMAP) of 134,776 single cells following extraction from PBMCs (7 Control, 5 NBEAL2, and 5 LRBA) and processed by scRNA-seq. A resolution of 1.8 allows us to identify 32 clusters based on the expression of specific markers and gene signatures. The cell subsets are represented and annotated with color and number (see legend at the bottom). **(B)** Heatmap of the average gene expression of CTLA-4, LRBA and NBEAL2 in the different T cell subsets of controls samples, after scRNA Seq analyses. Subsets are ranked from the highest NBEAL2 gene expression to the lowest. **(C)** Cluster biases observed in the sc RNA Seq experiment (performed once) after clustering in controls (N=7), NBEAL2 (N=5) and LRBA patients (N=5) PBMC samples. Each dot corresponds to an independent donor or patient. The line inside the box is the median value (50th percentile). Minima and maxima of the boxes correspond to 25th and 75th percentile. Whiskers marks the 10th and 90th percentile. Two-tailed p-values were determined with a nonparametric Mann-Whitney test. (*) p-value < 0.05; (**) p-value < 0.01; (***) p-value < 0.005 ; (****) p-value < 0.0001.

Supplementary Figure 9: single-cell RNA sequencing quality control – UMAP per sample

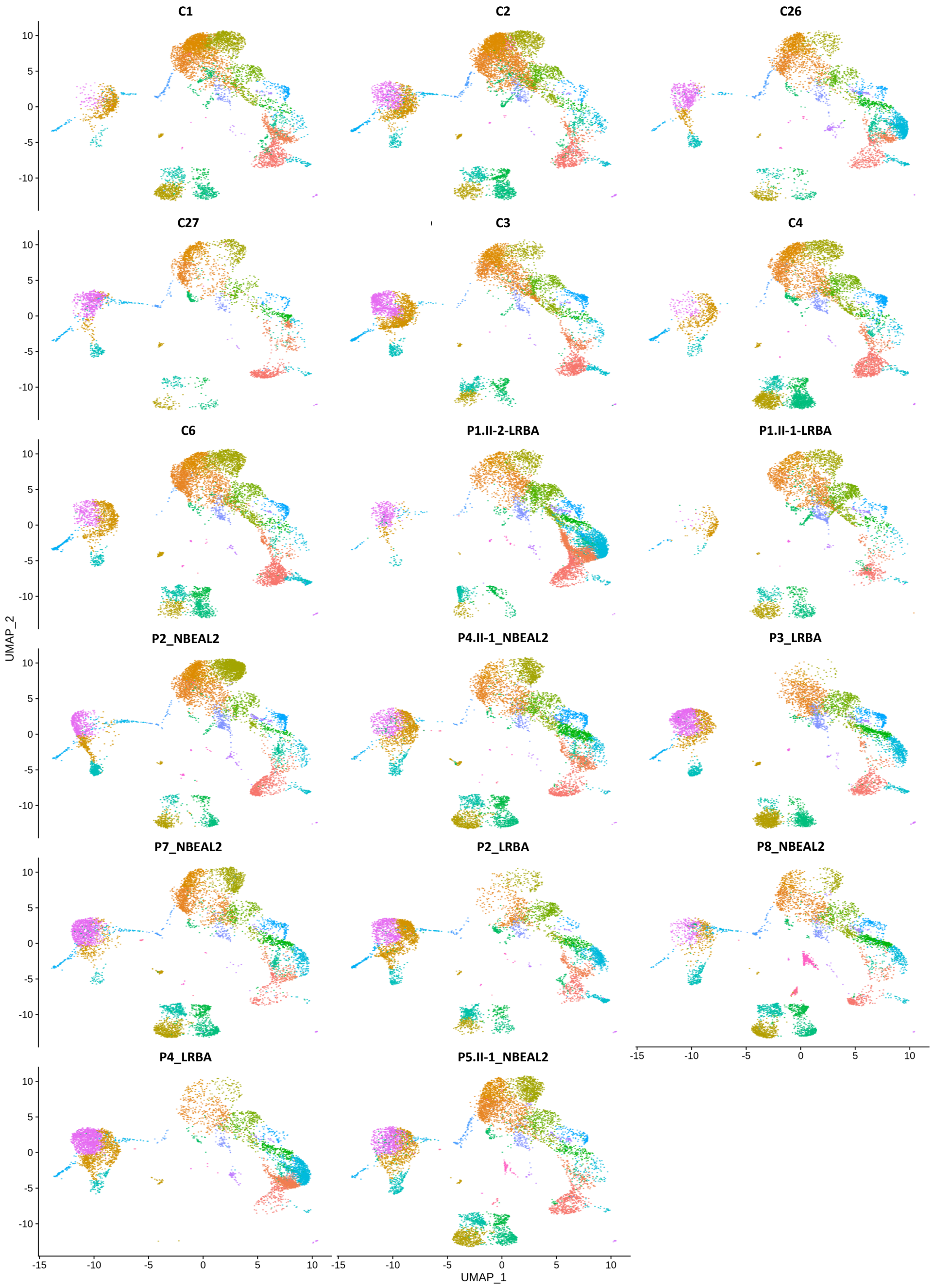

Supplementary Figure 9: single-cell RNA sequencing quality control – UMAP per sample. UMAP for each sample analyzed by single-cell RNA sequencing.

Supplementary Figure 10: sc RNA seq QC – markers used to perform cluster identification

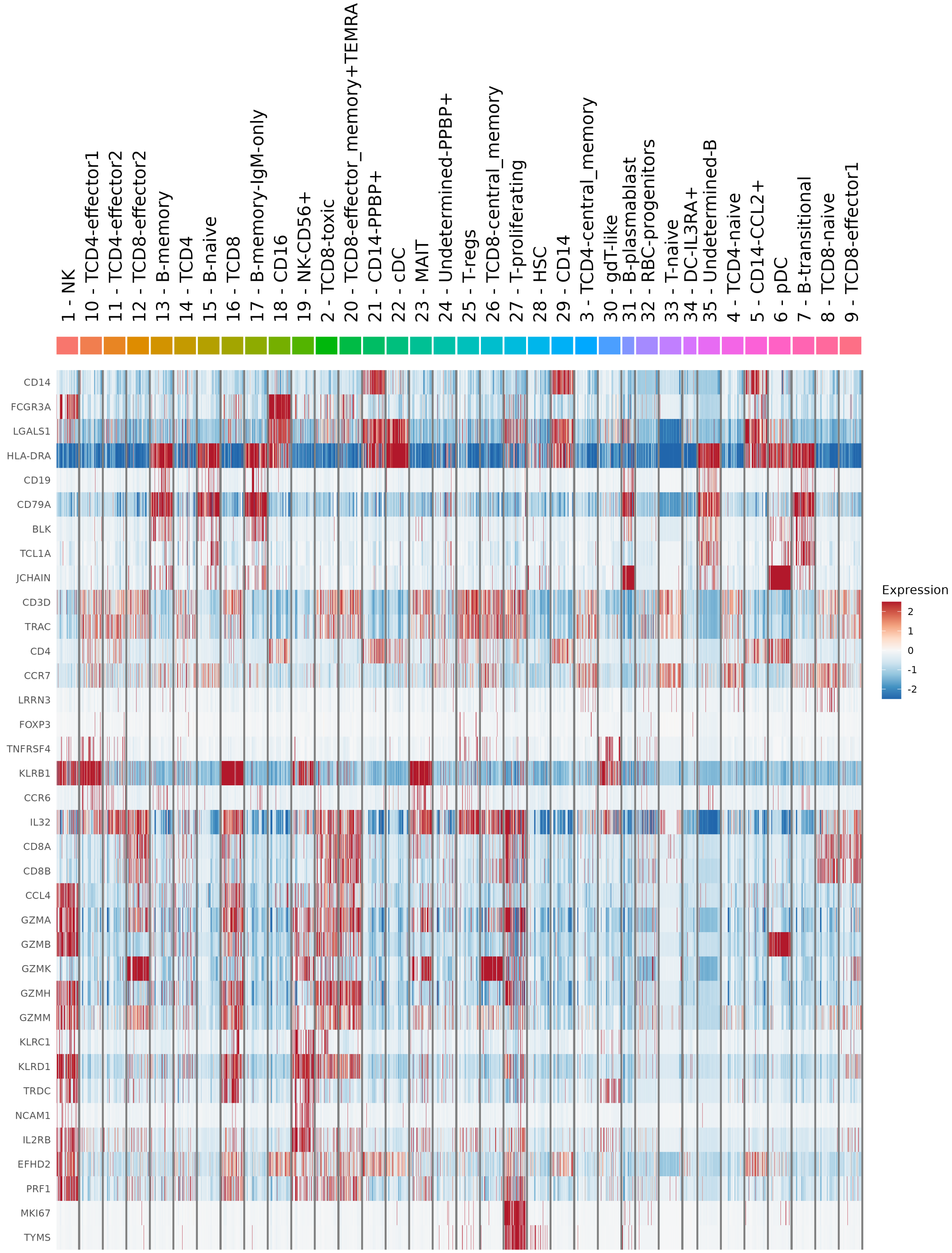

Supplementary Figure 10: sc RNA seq QC – markers used to perform cluster identification. Heatmap of intensities for each lineage marker used for cluster identification after unsupervised clustering for all samples.

Supplementary Figure 11: single-cell RNA sequencing quality control – percentage of mitochondrial genes per sample.

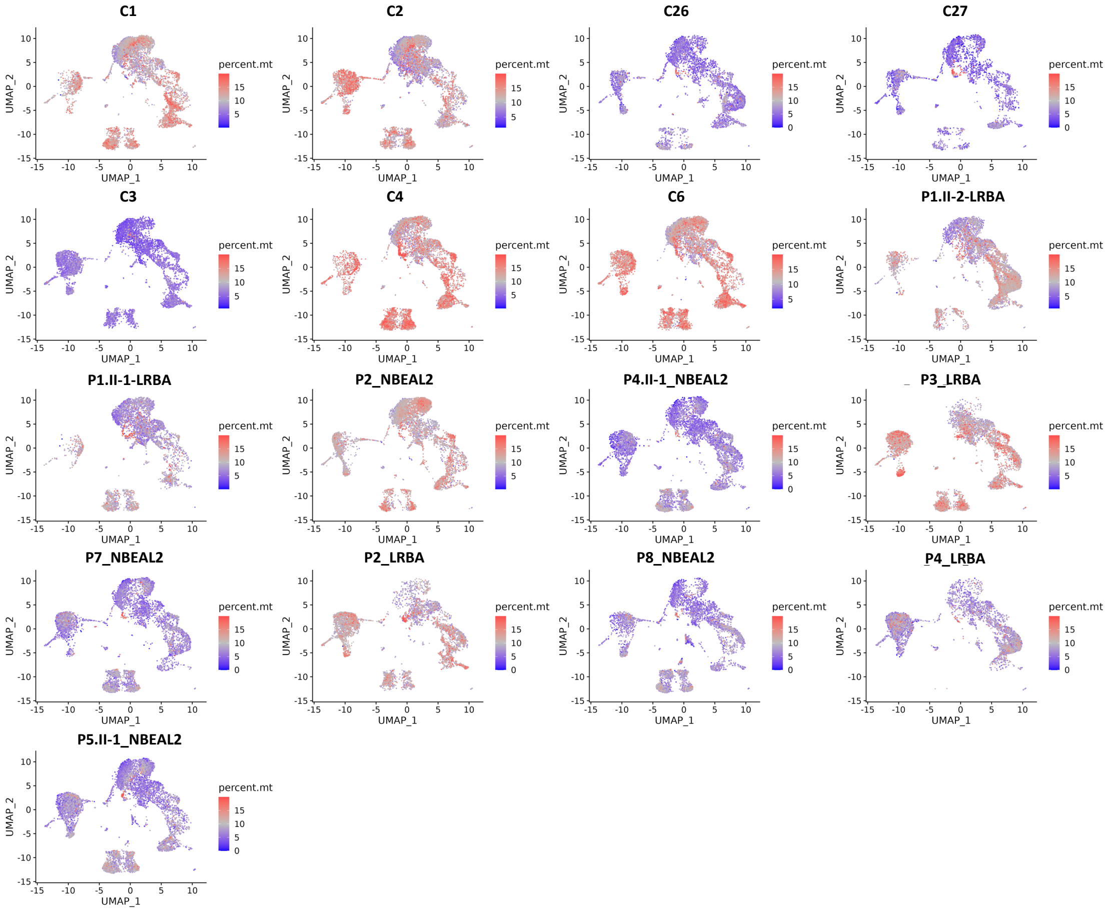

Supplementary Figure 11 – single-cell RNA sequencing quality control – percentage of mitochondrial genes per sample.

Supplementary Figure 12. IL-6 signaling pathway in LRBA deficient cells compared to NBEAL2 deficient cells – example of the CD4+ central memory subset.

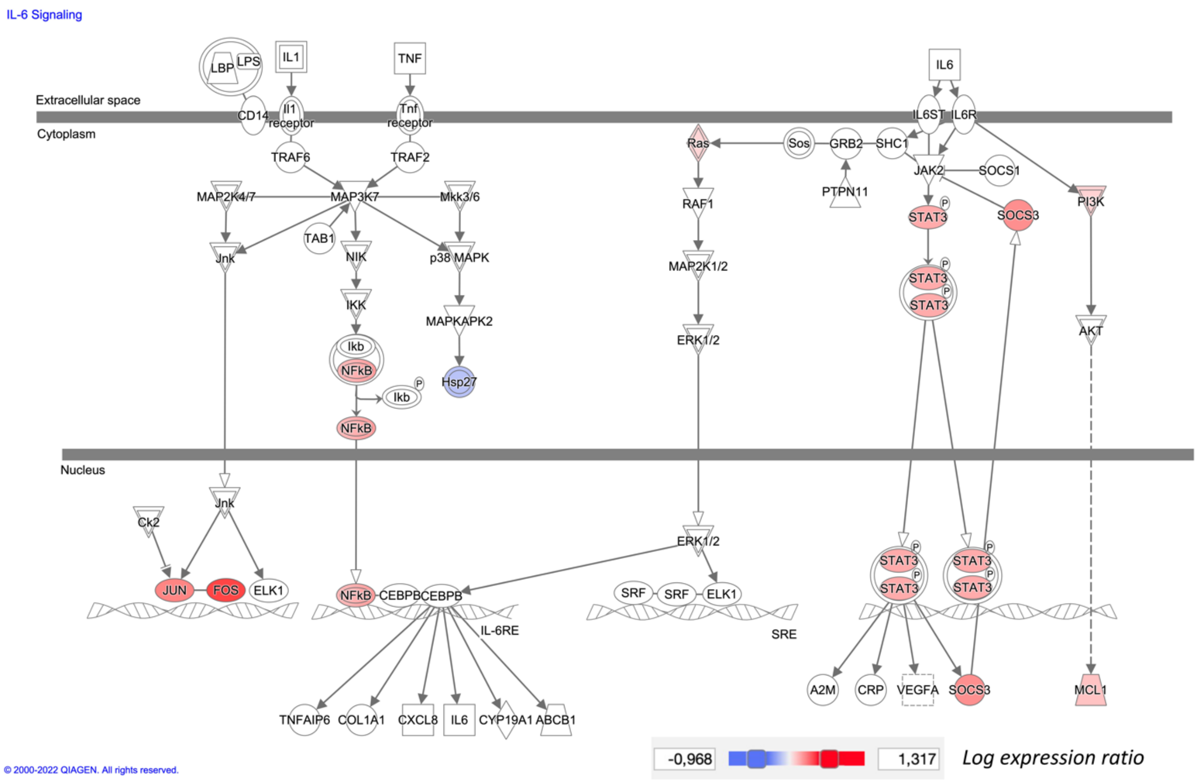

Supplementary Figure 12. IL-6 signaling pathway in LRBA deficient cells compared to NBEAL2 deficient cells – example of the CD4+ central memory subset. Illustration of the upregulation of IL-6 signaling pathway in LRBA deficient cells (here for the central memory CD4+ subset). This upregulation is found in other T cells subsets. The colors on the network correspond to the log ratio expression of the implicated genes. The network has been generated using the *Ingenuity Pathway Analysis* software.

Supplementary Table 1. List of detailed variants and publications where patients were already described.

Patient number	Affected allele	Var_ID1	Mutation 1	Var_ID2	Mutation 2	Var_ID3	Mutation 3	PubMed number of previous publications reporting the patient (Patient ID in the publication)
P1	homozygous	3_47044502_C_T	p.R1839C	3_47044502_C_T	p.R1839C	NA	NA	21765412 (GPS.5)
P2	homozygous	3_47042783_ATGC_A	p.L1501X	3_47042783_ATGC_A	p.L1501X	NA	NA	26472737 (P6), 33496751 (Patient H)
P3	homozygous	3_47037434_A_T	p.I682F	3_47037434_A_T	p.I682F	NA	NA	Never described.<br>Of note, the same variant was previously described: 21765411 (patient B.II.3) and 32693407 (family 21).<br>However, to our knowledge, the patient descibed in our manuscript was never described.
P4.II-1	compound heterozygous	3_47037233_A_T	p.E643V	3_47046466_C_T	p.P2100L	NA	NA	21765411 (patient C.II.4b), 32693407 (family 20), 33496751 (Patient B)
P4.II-2	compound heterozygous	3_47037233_A_T	p.E643V	3_47046466_C_T	p.P2100L	NA	NA	21765411 (Patient C.II.4a), 32693407 (family 20), 31502501
P4.II-3	compound heterozygous	3_47037233_A_T	p.E643V	3_47046466_C_T	p.P2100L	NA	NA	21765411 (Patient C.II.3), 32693407 (family 20), 33496751 (Patient A)
P5.II-1	homozygous	3_47043514_TG_T	p.R1631Gfs*3	3_47043514_TG_T	p.R1631Gfs*3	NA	NA	32693407 (patient P22.1), 33496751 (Patient G)
P5.II-2	homozygous	3_47043514_TG_T	p.R1631Gfs*3	3_47043514_TG_T	p.R1631Gfs*3	NA	NA	32693407 (patient P22.2)
P6	compound heterozygous	3_47039656_T_C	p.Y1020H	3_47038748_G_A	c.2650-1G>A	3_47047939_G_A	p.E2378E	32693407 (patient P31), 33496751 (patient F)
P7	compound heterozygous	3_47047440_C_T	p.S2269L	3_47049615_G_A	p.G2553E	3_47030447_A_G	p.I86V	21765411 (patient D.II.3), 32693407 (patient P24), 33496751 (Patient C)
P8	compound heterozygous	3_47046986_CT_C	p.C2190Xfs*23	3_47050068_T_C	p.L2646P	NA	NA	32693407 (patient P25), 33496751 (patient E)
P9.II-1	homozygous	3_47049139_GAC_G	p.T2487fs*16	3_47049139_GAC_G	p.T2487X	p.T2487fs*16	NA	Never described
P9.II-2	homozygous	3_47049139_GAC_G	p.T2487fs*16	3_47049139_GAC_G	p.T2487X	p.T2487fs*16	NA	Never described

Supplementary Table 2. Comparison between our mass spectrometry data and the data from Mayer et al., *Blood, 2018* and Lo et al., *Blood,* 2020. In blue, proteins found by Mayer et al., Blood, 2018 and in our interactome. In orange, SEC22B, the protein described to interact with NBEAL2 by Lo et al., Blood, 2020.

Data from Mayer et al., Blood, 2018 and Lo et al., Blood, 2020					Our data				
**Gene name**	**Uniprot AC**	**Description**	**Validation (Reverse IP) by Mayer et al**	**Protein Class**	**Ratio ctrls / P6**	**Ratio ctrls / P7**	**corrected ratio mean ctrsl/P6**	**corrected ratio mean ctrls/P7**	**mean intensities controls**
NBEAL2	H7C3Y7	Neurobeachin-like protein 2 (Fragment)	NA (Bait)	phospholipid binding	665,43	19,97	665,43	19,97	**1,24E+11**
HSPB1	C9J3N8	Heat shock protein beta-1	not tested	enzymes/enzyme modulators	#DIV/0!	1,33	96,05	1,33	**1,12E+07**
TUBB4A	P04350	Tubulin beta-4A chain	not tested	cytoskeletal proteins	66,36	0,78	66,36	0,78	**3,87E+08**
VAC14	Q08AM6	Protein VAC14 homolog	Interaction confirmed	receptor activity/receptor activity	#DIV/0!	1,58	18,65	1,58	**2,18E+06**
STAU1	Q5JW30	Double-stranded RNA-binding protein Staufen homolog 1	not tested	enzymes/enzyme modulators	#DIV/0!	2,49	12,47	2,49	**1,46E+06**
RPS8	Q5JR95	40S ribosomal protein S8	not tested	nucleic acid binding	9,28	1,50	9,28	1,50	**9,70E+06**
SEC16A	J3KNL6	Protein transport protein Sec16A	Interaction confirmed	protein binding	#DIV/0!	#DIV/0!	8,09	8,09	**9,46E+05**
GIGYF2	C9JW88	ERQ amino acid-rich with GYF domain-containing protein 2 (Fragmen	not tested	nucleic acid binding	7,62	1,28	7,62	1,28	**9,74E+06**
BAG2	O95816	BAG family molecular chaperone regulator 2	not tested	chaperones/heat shock prtoteins	7,15	#DIV/0!	7,15	45,10	**5,28E+06**
YTHDF2	Q9Y5A9	YTH domain family protein 2	not tested	nucleic acid binding	5,41	0,80	5,41	0,80	**4,98E+06**
FAM120A	H7C0T0	Constitutive coactivator of PPAR-gamma-like protein 1 (Fragment)	not tested	nucleic acid binding	5,26	12,97	5,26	12,97	**9,27E+06**
LARP1	E5RH50	La-related protein 1 (Fragment)	not tested	protein translation	5,18	2,69	5,18	2,69	**1,45E+07**
EIF4G1	G5E9S1	Eukaryotic translation initiation factor 4 gamma 1	not tested	protein translation	4,82	2,55	4,82	2,55	**2,41E+07**
OGT	O15294	cetylglucosamine--peptide N-acetylglucosaminyltransferase 110 kDa	not tested	enzymes/enzyme modulators	4,80	1,08	4,80	1,08	**6,02E+06**
POLR2B	C9J4M6	DNA-directed RNA polymerase	not tested	enzymes/enzyme modulators	4,70	3,35	4,70	3,35	**1,47E+07**
ATXN2L	Q8WWM7-6	Isoform 6 of Ataxin-2-like protein	not tested	nucleic acid binding	4,43	4,99	4,43	4,99	**3,68E+06**
UPF1	Q92900-2	Isoform 2 of Regulator of nonsense transcripts 1	not tested	nucleic acid binding	4,37	3,35	4,37	3,35	**6,44E+07**
DDX17	G5E9L5	DEAD (Asp-Glu-Ala-Asp) box polypeptide 17, isoform CRA_a	not tested	transcription factors/cofactors	3,70	0,44	3,70	0,44	**2,41E+08**
RPL24	C9JXB8	60S ribosomal protein L24	not tested	nucleic acid binding	3,67	1,52	3,67	1,52	**7,92E+07**
NONO	Q15233	Non-POU domain-containing octamer-binding protein	not tested	transcription factors/cofactors	3,58	1,06	3,58	1,06	**5,85E+07**
ZC3HAV1	Q7Z2W4-2	Isoform 2 of Zinc finger CCCH-type antiviral protein 1	not tested	enzymes/enzyme modulators	3,47	1,49	3,47	1,49	**6,07E+07**
TUBG1	K7EKE5	Tubulin gamma-1 chain	not tested	cytoskeletal proteins	3,45	1,65	3,45	1,65	**2,42E+07**
DNAJA1	P31689	DnaJ homolog subfamily A member 1	not tested	chaperones/heat shock prtoteins	3,33	1,62	3,33	1,62	**4,40E+07**
SLTM	H0YKH2	SAFB-like transcription modulator (Fragment)	not tested	nucleic acid binding	3,27	1,46	3,27	1,46	**2,09E+06**
CRLF3	Q8IUI8-2	Isoform 2 of Cytokine receptor-like factor 3	not tested	protein dimerization	3,22	0,90	3,22	0,90	**1,91E+07**

SORD	H0YLA4	Sorbitol dehydrogenase	not tested	enzymes/enzyme modulators	3,06	1,25	3,06	1,25	**9,50E+06**
BAG6	B0UX83	HLA-B associated transcript 3	not tested	chaperones/heat shock prtoteins	2,95	4,22	2,95	4,22	**2,25E+07**
HUWE1	H0Y5W0	E3 ubiquitin-protein ligase HUWE1 (Fragment)	not tested	enzymes/enzyme modulators	2,87	1,07	2,87	1,07	**3,73E+07**
C14orf166	H0YJB9	UPF0568 protein C14orf166 (Fragment)	not tested	protein dimerization	2,80	1,35	2,80	1,35	**1,14E+07**
SHMT1	B4DPM9	Serine hydroxymethyltransferase	not tested	enzymes/enzyme modulators	2,80	1,23	2,80	1,23	**1,17E+07**
PABPC4	Q13310-2	Isoform 2 of Polyadenylate-binding protein 4	not tested	nucleic acid binding	2,68	0,92	2,68	0,92	**3,79E+06**
LARP4B	H0Y4V9	La-related protein 4B (Fragment)	not tested	nucleic acid binding	2,67	1,62	2,67	1,62	**2,49E+06**
DNAJA2	O60884	DnaJ homolog subfamily A member 2	not tested	chaperones/heat shock prtoteins	2,52	0,96	2,52	0,96	**4,03E+06**
UBAP2L	H0Y5H6	Ubiquitin-associated protein 2-like (Fragment)	not tested	nucleic acid binding	2,49	3,13	2,49	3,13	**2,21E+06**
APOL2	Q9BQE5	Apolipoprotein L2	not tested	receptor activity/receptor activity	2,44	1,17	2,44	1,17	**1,53E+07**
SEC22B	O75396	Vesicle-trafficking protein SEC22b	Interaction confirmed	protein binding	2,43	1,61	2,43	1,61	**3,71E+07**
AIP	O00170	AH receptor-interacting protein	not tested	chaperones/heat shock prtoteins	2,41	2,18	2,41	2,18	**1,17E+08**
AGO2	Q9UKV8	Protein argonaute-2	not tested	protein translation	2,38	1,83	2,38	1,83	**6,36E+06**
CCT8	G5E9B2	Chaperonin containing TCP1, subunit 8 (Theta), isoform CRA_a	not tested	chaperones/heat shock prtoteins	2,37	1,11	2,37	1,11	**6,88E+08**
RFC5	P40937	Replication factor C subunit 5	not tested	enzymes/enzyme modulators	2,22	1,51	2,22	1,51	**2,89E+07**
CAPRIN1	E9PLA9	Caprin-1 (Fragment)	not tested	nucleic acid binding	2,20	1,26	2,20	1,26	**1,91E+07**
DNAJC7	K7ELJ8	DnaJ homolog subfamily C member 7	not tested	chaperones/heat shock prtoteins	2,11	1,00	2,11	1,00	**1,24E+07**
PSMC2	B7Z5E2	26S protease regulatory subunit 7	not tested	enzymes/enzyme modulators	2,06	0,89	2,06	0,89	**8,34E+07**
HNRNPA0	Q13151	Heterogeneous nuclear ribonucleoprotein A0	not tested	enzymes/enzyme modulators	2,05	1,36	2,05	1,36	**2,52E+07**
RANGAP1	B0QYT6	Ran GTPase activating protein 1 (Fragment)	not tested	enzymes/enzyme modulators	1,98	1,14	1,98	1,14	**1,67E+07**
RUVBL2	Q9Y230	RuvB-like 2	not tested	chaperones/heat shock prtoteins	1,94	1,27	1,94	1,27	**2,61E+08**
NTPCR	Q9BSD7	Cancer-related nucleoside-triphosphatase	not tested	enzymes/enzyme modulators	1,91	1,36	1,91	1,36	**1,30E+07**
HSPA1B	F8VZJ4	Heat shock 70 kDa protein 1A/1B	Interaction not replicated	chaperones/heat shock prtoteins	1,91	1,10	1,91	1,10	**2,95E+08**
PABPC1	P11940	Polyadenylate-binding protein 1	not tested	protein translation	1,87	1,03	1,87	1,03	**1,08E+08**
SERBP1	Q8NC51-4	Isoform 4 of Plasminogen activator inhibitor 1 RNA-binding protein	not tested	nucleic acid binding	1,79	1,07	1,79	1,07	**8,10E+07**
RPS13	E9PS50	40S ribosomal protein S13 (Fragment)	not tested	nucleic acid binding	1,76	0,77	1,76	0,77	**5,83E+07**
RUVBL1	E7ETR0	RuvB-like 1	not tested	enzymes/enzyme modulators	1,76	1,55	1,76	1,55	**1,72E+08**
HNRNPAB	D6R9P3	Heterogeneous nuclear ribonucleoprotein A/B	not tested	transcription factors/cofactors	1,75	0,98	1,75	0,98	**6,47E+07**
FARSA	B4E363	Phenylalanine--tRNA ligase alpha subunit	not tested	enzymes/enzyme modulators	1,74	1,01	1,74	1,01	**2,31E+08**
NUDT21	H3BV41	Cleavage and polyadenylation-specificity factor subunit 5 (Fragment)	not tested	enzymes/enzyme modulators	1,69	1,68	1,69	1,68	**2,41E+07**
TUBB	F8VW92	Tubulin beta chain	Interaction not replicated	cytoskeletal proteins	1,68	1,06	1,68	1,06	**6,25E+09**

RTCB	Q9Y3I0	tRNA-splicing ligase RtcB homolog	not tested	nucleic acid binding	1,67	1,00	1,67	1,00	**8,03E+07**
RPL10A	P62906	60S ribosomal protein L10a	not tested	nucleic acid binding	1,66	0,80	1,66	0,80	**8,26E+07**
POLR2E	P19388	DNA-directed RNA polymerases I, II, and III subunit RPABC1	not tested	enzymes/enzyme modulators	1,66	1,00	1,66	1,00	**5,54E+06**
MTHFD1	G3V3L6	C-1-tetrahydrofolate synthase, cytoplasmic	not tested	enzymes/enzyme modulators	1,63	0,99	1,63	0,99	**1,33E+09**
TARDBP	B4DJ45	TAR DNA-binding protein 43	not tested	transcription factors/cofactors	1,63	1,25	1,63	1,25	**5,83E+07**
TUBB2A	Q13885	Tubulin beta-2A chain	not tested	cytoskeletal proteins	1,53	0,49	1,53	0,49	**1,12E+07**
TUBB4B	P68371	Tubulin beta-4B chain	Interaction not replicated	cytoskeletal proteins	1,53	1,02	1,53	1,02	**2,23E+10**
GPN1	B5MBZ5	GPN-loop GTPase 1	not tested	enzymes/enzyme modulators	1,50	1,30	1,50	1,30	**3,12E+06**
HNRNPD	D6RF44	Heterogeneous nuclear ribonucleoprotein D0 (Fragment)	not tested	transcription factors/cofactors	1,35	1,00	1,35	1,00	**3,14E+08**
CCAR2	G3V119	DBIRD complex subunit KIAA1967	not tested	enzymes/enzyme modulators	1,28	1,12	1,28	1,12	**1,70E+08**
UBC	F5H265	Polyubiquitin-C (Fragment)	Interaction not replicated	enzymes/enzyme modulators	1,26	1,07	1,26	1,07	**1,25E+09**
ILF3	G5E9M5	Interleukin enhancer binding factor 3, 90kDa, isoform CRA_b	not tested	nucleic acid binding	1,23	0,70	1,23	0,70	**3,19E+07**
HNRNPA3	P51991	Heterogeneous nuclear ribonucleoprotein A3	not tested	nucleic acid binding	1,23	1,11	1,23	1,11	**1,61E+08**
POLR1C	O15160-2	Isoform 2 of DNA-directed RNA polymerases I and III subunit RPAC1	not tested	enzymes/enzyme modulators	1,17	1,04	1,17	1,04	**8,52E+06**
ALYREF	Q86V81	THO complex subunit 4	not tested	nucleic acid binding	1,12	0,77	1,12	0,77	**1,49E+08**
PABPN1	Q86U42-2	Isoform 2 of Polyadenylate-binding protein 2	not tested	nucleic acid binding	1,03	0,98	1,03	0,98	**2,13E+07**
RALY	Q5QPRMN0A	ein, autoantigenic (HnRNP-associated with lethal yellow homolog (M	not tested	nucleic acid binding	1,02	1,97	1,02	1,97	**1,82E+07**
HNRNPR	Q2L7G6	Heterogeneous nuclear ribonucleoprotein R	not tested	nucleic acid binding	1,02	0,73	1,02	0,73	**2,39E+07**
LARP4	G5E976	La ribonucleoprotein domain family, member 4, isoform CRA_h	not tested	nucleic acid binding	0,95	0,56	0,95	0,56	**3,79E+05**
CPSF4	O95639	Cleavage and polyadenylation specificity factor subunit 4	not tested	nucleic acid binding	0,91	0,54	0,91	0,54	**5,95E+05**
RBBP7	E9PC52	Histone-binding protein RBBP7	not tested	nucleic acid binding	0,86	0,94	0,86	0,94	**2,32E+07**
RBMX	H0Y6E7	binding motif protein, X chromosome, N-terminally processed (Frag	not tested	nucleic acid binding	0,77	1,19	0,77	1,19	**6,20E+06**
CPSF1	Q10570	Cleavage and polyadenylation specificity factor subunit 1	not tested	enzymes/enzyme modulators	0,50	#DIV/0!	0,50	7,12	**8,33E+05**
TUBB6	Q9BUF5	Tubulin beta-6 chain	not tested	cytoskeletal proteins	0,03	0,25	0,03	0,25	**4,20E+05**
AKAP8	O43823	A-kinase anchor protein 8	not tested	enzymes/enzyme modulators	NA	NA	NA	NA	NA
AKAP8L	Q9ULX6-2	Isoform 2 of A-kinase anchor protein 8-like	not tested	enzymes/enzyme modulators	NA	NA	NA	NA	NA
ALDH7A1	P49419-2	Isoform 2 of Alpha-aminoadipic semialdehyde dehydrogenase	not tested	enzymes/enzyme modulators	NA	NA	NA	NA	NA
AMOT	Q4VCS5-2	Isoform 2 of Angiomotin	not tested	receptor activity/receptor activity	NA	NA	NA	NA	NA
ATXN2	H0YH87	Ataxin-2 (Fragment)	Interaction not replicated	receptor activity/receptor activity	NA	NA	NA	NA	NA
BAG4	O95429-2	Isoform 2 of BAG family molecular chaperone regulator 4	not tested	chaperones/heat shock prtoteins	NA	NA	NA	NA	NA

CAMKC2aAlci	D6RHX9	m/calmodulin-dependent protein kinase type II subunit alpha (Fragmen2t7)	not tested	enzymes/enzyme modulators	NA	NA	NA	NA	NA
CDC42EP1	Q00587-2	Isoform 2 of Cdc42 effector protein 1	not tested	enzymes/enzyme modulators	NA	NA	NA	NA	NA
CHERP	Q8IWX8	Calcium homeostasis endoplasmic reticulum protein	not tested	ion channel binding	NA	NA	NA	NA	NA
CPEB4	H0YBG1	Cytoplasmic polyadenylation element-binding protein 4 (Fragment)	not tested	protein translation	NA	NA	NA	NA	NA
CPSF7	J3QT54	Cleavage and polyadenylation-specificity factor subunit 7 (Fragment)	not tested	nucleic acid binding	NA	NA	NA	NA	NA
DNAJB6	C9JB42	DnaJ homolog subfamily B member 6 (Fragment)	not tested	chaperones/heat shock prtoteins	NA	NA	NA	NA	NA
DOCK7	H0Y7L2	Dedicator of cytokinesis protein 7 (Fragment)	Interaction confirmed	enzymes/enzyme modulators	NA	NA	NA	NA	NA
EIF4G3	B1AN89	Eukaryotic translation initiation factor 4 gamma 3	not tested	protein translation	NA	NA	NA	NA	NA
ELAVL2	B1AM48	(Embryonic lethal, abnormal vision, Drosophila)-like 2 (Hu antigen B) (Fragm82e.n7t0)	not tested	nucleic acid binding	NA	NA	NA	NA	NA
FIP1L1	G3XAD6	FIP1 like 1 (S. cerevisiae), isoform CRA_d	not tested	nucleic acid binding	NA	NA	NA	NA	NA
FMR1	A8MQB8	Fragile X mental retardation protein 1	not tested	protein dimerization	NA	NA	NA	NA	NA
FXR2	I3L1Z2	Fragile X mental retardation syndrome-related protein 2 (Fragment)	not tested	protein dimerization	NA	NA	NA	NA	NA
GTF3C5	Q5T7U1	General transcription factor 3C polypeptide 5	not tested	nucleic acid binding	NA	NA	NA	NA	NA
HAX1	E9PIQ7	HCLS1-associated protein X-1	not tested	interleukin-1 binding	NA	NA	NA	NA	NA
HELZ	J3QS41	Probable helicase with zinc finger domain	not tested	enzymes/enzyme modulators	NA	NA	NA	NA	NA
IGF2BP2	F8W930	Insulin-like growth factor 2 mRNA-binding protein 2	not tested	protein translation	NA	NA	NA	NA	NA
IGF2BP3	F8WD15	Insulin-like growth factor 2 mRNA-binding protein 3	not tested	protein translation	NA	NA	NA	NA	NA
LSM12	K7ELG9	Protein LSM12 homolog	not tested	unclassified	NA	NA	NA	NA	NA
MOV10	Q5JR04	Mov10, Moloney leukemia virus 10, homolog (Mouse)	not tested	nucleic acid binding	NA	NA	NA	NA	NA
NCOA1	B5MCN7	Nuclear receptor coactivator 1	not tested	enzymes/enzyme modulators	NA	NA	NA	NA	NA
NCOR1	O75376-2	Isoform 2 of Nuclear receptor corepressor 1	not tested	transcription factors/cofactors	NA	NA	NA	NA	NA
NUFIP2	Q7Z417	Nuclear fragile X mental retardation-interacting protein 2	Interaction not replicated	nucleic acid binding	NA	NA	NA	NA	NA
PIKFYVE	E9PDH4	1-phosphatidylinositol 3-phosphate 5-kinase (Fragment)	not tested	enzymes/enzyme modulators	NA	NA	NA	NA	NA
PRRC2A	C9J1F6	Protein PRRC2A	not tested	nucleic acid binding	NA	NA	NA	NA	NA
PUM1	E9PR38	Pumilio homolog 1	not tested	nucleic acid binding	NA	NA	NA	NA	NA
QKI	F5GYT7	Protein quaking	not tested	nucleic acid binding	NA	NA	NA	NA	NA
RANBP2	F8WBP7	Putative peptidyl-prolyl cis-trans isomerase	not tested	enzymes/enzyme modulators	NA	NA	NA	NA	NA
RBM14	Q96PK6	RNA-binding protein 14	not tested	transcription factors/cofactors	NA	NA	NA	NA	NA
RBM7	G3V1T9	RNA binding motif protein 7, isoform CRA_a	not tested	nucleic acid binding	NA	NA	NA	NA	NA
RBMS1	E7EPF2	RNA-binding motif, single-stranded-interacting protein 1 (Fragment)	not tested	nucleic acid binding	NA	NA	NA	NA	NA

SRBD1	B7Z6X7	S1 RNA-binding domain-containing protein 1	not tested	nucleic acid binding	NA	NA	NA	NA	NA
STAU2	G5EA18	Double-stranded RNA-binding protein Staufen homolog 2	not tested	nucleic acid binding	NA	NA	NA	NA	NA
SUGP2	E7ETX7	SURP and G-patch domain-containing protein 2	not tested	nucleic acid binding	NA	NA	NA	NA	NA
THRAP3	Q9Y2W1	Thyroid hormone receptor-associated protein 3	not tested	transcription factors/cofactors	NA	NA	NA	NA	NA
TNRC6A	H3BTQ1	Trinucleotide repeat-containing gene 6A protein (Fragment)	not tested	nucleic acid binding	NA	NA	NA	NA	NA
TRO	B1AKE8	Trophinin (Fragment)	not tested	protein binding	NA	NA	NA	NA	NA
TUBA1A	G3V1U9	Tubulin alpha-1A chain	Interaction not replicated	cytoskeletal proteins	NA	NA	NA	NA	NA
TUBA3C	Q13748	Tubulin alpha-3C/D chain	not tested	cytoskeletal proteins	NA	NA	NA	NA	NA
UBR5	E7EMW7	E3 ubiquitin-protein ligase UBR5	not tested	enzymes/enzyme modulators	NA	NA	NA	NA	NA
YBX3	P16989	DNA-binding protein A	not tested	enzymes/enzyme modulators	NA	NA	NA	NA	NA
YLPM1	H0YIQ2	YLP motif-containing protein 1 (Fragment)	not tested	nucleic acid binding	NA	NA	NA	NA	NA
ZFR	Q96KR1	Zinc finger RNA-binding protein	not tested	nucleic acid binding	NA	NA	NA	NA	NA
ZNF318	Q5VUA4	Zinc finger protein 318	not tested	nucleic acid binding	NA	NA	NA	NA	NA
ZNF326	Q5BKZ1	DBIRD complex subunit ZNF326	not tested	enzymes/enzyme modulators	NA	NA	NA	NA	NA
CDC73	Q6P1J9	Parafibromin	not tested	enzymes/enzyme modulators	NA	NA	NA	NA	NA
HNRNPA3	E7EWI9	Heterogeneous nuclear ribonucleoprotein A3	not tested	nucleic acid binding	NA	NA	NA	NA	NA
F8W810	F8W810	unknwon function	not tested	enzymes/enzyme modulators	NA	NA	NA	NA	NA

Supplementary Table 3. Single-cell RNA sequencing quality controls. For each sample, before and after filtering, the following data are compiled: number of cells (nCount), median number of genes detected in all cells of the sample (nFeature_median), median number of UMI detected in all cells of the sample (nCount_median), median percentage of mitochondrial content in all cells of the sample (MT_median), median percentage of ribosomal content in all cells of the sample (Ribo_median), the cell viability after thawing and the estimate fraction of contaminants following SoupX analysis.

Sample ID	Status	nCells	nFeature_ median	nCount_ median	MT_ median	Ribo_ median	Cell viability after thawing	SoupX estimate contamination fractions
C1	Pre-filtered	8874	1031	3600	10	43	96,3%	NA
C1	Filtered	7305	1082	4023	10	47	NA	5,3
C26	Pre-filtered	7311	1558	4675	5	30	98,4%	NA
C26	Filtered	5435	1747	5509	5	31	NA	0,9
C27	Pre-filtered	5299	1459	4790	4	28	98,3%	NA
C27	Filtered	3816	1688	5932	4	37	NA	3,1
C2	Pre-filtered	11016	912	2904	10	42	97,5%	NA
C2	Filtered	9074	961	3160	9	45	NA	5,4
C3	Pre-filtered	11787	554	1530	5	40	97,0%	NA
C3	Filtered	7171	651	2034	5	46	NA	5,2
C4	Pre-filtered	11008	1016	2590	14	27	96,4%	NA
C4	Filtered	7436	1125	3120	12	34	NA	5
C6	Pre-filtered	10740	982	2816	13	31	95,5%	NA
C6	Filtered	8320	1055	3185	12	36	NA	2,1
P1.II-2_LRBA	Pre-filtered	8822	1284	3255	9	29	97,1%	NA
P1.II-2_LRBA	Filtered	8052	1313	3370	9	29	NA	1,8
P1.II-1_LRBA	Pre-filtered	5763	1236	4105	7	41	96,5%	NA
P1.II-1_LRBA	Filtered	4805	1296	4471	7	43	NA	2,2
P2_NBEAL2	Pre-filtered	8714	1128	3470	10	37	98,4%	NA
P2_NBEAL2	Filtered	7835	1168	3643	10	38	NA	1,4
P4.II-1_NBEAL2	Pre-filtered	9661	1523	4857	5	35	98,4%	NA
P4.II-1_NBEAL2	Filtered	7535	1673	5553	5	37	NA	3,1
P3_LRBA	Pre-filtered	8551	1101	2888	11	28	96,1%	NA
P3_LRBA	Filtered	7289	1163	3120	10	30	NA	1,3
P7_NBEAL2	Pre-filtered	9270	1292	4288	6	37	99%	NA
P7_NBEAL2	Filtered	6979	1461	4978	6	41	NA	5,2
P2_LRBA	Pre-filtered	7084	1389	4062	10	25	94,8%	NA
P2_LRBA	Filtered	6028	1508	4720	9	27	NA	1
P8_NBEAL2	Pre-filtered	10895	961	4676	4	18	94,0%	NA
P8_NBEAL2	Filtered	5851	1514	4907	6	36	NA	5,4

P4_LRBA	Pre-filtered	8149	1581	4363	6	24	95,1%	NA
P4_LRBA	Filtered	6186	1762	5058	6	24	NA	0,3
P5.II-1_NBEAL2	Pre-filtered	10581	1331	4541	6	36	95,1%	NA
P5.II-1_NBEAL2	Filtered	7928	1494	5163	6	41	NA	2,4

Supplementary Table 4. Analyses and comparison of proteomics and transcriptomics data in activated T cells from healhy donors and NBEAL2 deficient patients. After proteome analyses, 21 proteins are expressed in the tested controls but not in the NBEAL2 deficient patients (green). 23 proteins are expressed in NBEAL2 deficient patient samples but not in healthy donors (in yellow). 4 proteins have different protein expression in NBEAL2 deficient patients compared to controls (in blue). The mean normalized data count of the transcripts coding for the identified proteins have been reported in the last columns of the table. The ratio between normalized data count of the NBEAL2 deficient patients versus the controls have been calculated.

Proteome data									Bulk RNA Seq data			
Protein IDs	Protein names	HUGO-ID	FC	Claspatient.p val	CtrlS5	CtrlS7	patient_ P7	patient_ P6	GENEID	Mean data count controls	Mean data count NBEAL2 patients	ratio patients / controls
Q8TF46	DIS3-like exonuclease 1	DIS3L	**-217,6**	**0,00570998**	2,76E+08	1,83E+08	0,00E+00	0,00E+00	ENSG00000166938	1734,7	1795,8	1,04
Q6ZNJ1	Neurobeachin-like protein 2	NBEAL2	**-83,3**	**0,02508958**	5,38E+07	1,25E+08	0,00E+00	0,00E+00	ENSG00000160796	18351,6	17203,6	0,94
Q9P1Z2	Calcium-binding and coiled-coil domain-containing protein 1	CALCOCO1	**-75,2**	**0,00211636**	6,98E+07	8,83E+07	0,00E+00	0,00E+00	ENSG00000012822	2861,0	3004,7	1,05
O60784	Target of Myb protein 1	TOM1	**-66,9**	**0,00675784**	5,60E+07	8,51E+07	0,00E+00	0,00E+00	ENSG00000100284	1113,9	1093,1	0,98
Q01543	Friend leukemia integration 1 transcription factor	FLI1	**-40,0**	**0,00503057**	3,50E+07	4,92E+07	0,00E+00	0,00E+00	ENSG00000151702	8222,4	8377,0	1,02
Q86X10	Ral GTPase-activating protein subunit beta	RALGAPB	**-37,1**	**0,00283921**	4,39E+07	3,41E+07	0,00E+00	0,00E+00	ENSG00000170471	3490,5	3442,7	0,99
Q9NRR5	Ubiquilin-4	UBQLN4	**-35,8**	**2,89E-06**	3,77E+07	3,74E+07	0,00E+00	0,00E+00	ENSG00000160803	2665,2	2693,3	1,01
Q676U5	Autophagy-related protein 16-1	ATG16L1	**-35,1**	**0,00012094**	3,78E+07	3,59E+07	0,00E+00	0,00E+00	ENSG00000085978	2221,6	2235,7	1,01
P53701	Cytochrome c-type heme lyase	HCCS	**-31,5**	**0,03696723**	4,87E+07	1,92E+07	0,00E+00	0,00E+00	ENSG00000004961	676,5	682,1	1,01
P48553	Trafficking protein particle complex subunit 10	TRAPPC10	**-29,4**	**0,00061757**	2,91E+07	3,26E+07	0,00E+00	0,00E+00	ENSG00000160218	3471,2	3421,1	0,99
O00330	Pyruvate dehydrogenase protein X component, mitochondrial	PDHX	**-25,5**	**0,00606176**	2,22E+07	3,15E+07	0,00E+00	0,00E+00	ENSG00000110435	780,3	765,5	0,98
Q9BUF5	Tubulin beta-6 chain	TUBB6	**-22,9**	**0,00013052**	2,34E+07	2,46E+07	0,00E+00	0,00E+00	ENSG00000176014	235,1	207,1	0,88
Q6NYC1	Bifunctional arginine demethylase and lysyl-hydroxylase JMJD6	JMJD6	**-22,4**	**0,01673386**	1,71E+07	3,04E+07	0,00E+00	0,00E+00	ENSG00000070495	1723,9	1729,8	1,00
Q01581	Hydroxymethylglutaryl-CoA synthase, cytoplasmic	HMGCS1	**-21,4**	**0,03604647**	1,37E+07	3,23E+07	0,00E+00	0,00E+00	ENSG00000112972	2221,3	2181,7	0,98
P13797	Plastin-3	PLS3	**-20,3**	**0,02475765**	2,88E+07	1,44E+07	0,00E+00	0,00E+00	ENSG00000102024	27,3	13,8	0,50
Q15785	Mitochondrial import receptor subunit TOM34	TOMM34	**-14,1**	**0,00356799**	1,66E+07	1,31E+07	0,00E+00	0,00E+00	ENSG00000025772	1206,9	1303,3	1,08
Q9NV88	Integrator complex subunit 9	INTS9	**-13,1**	**0,00716696**	1,15E+07	1,60E+07	0,00E+00	0,00E+00	ENSG00000104299	1174,9	1198,6	1,02
Q6NUQ4	Transmembrane protein 214	TMEM214	**-12,3**	**0,00020494**	1,32E+07	1,25E+07	0,00E+00	0,00E+00	ENSG00000119777	4049,4	4068,9	1,00
P81605	Dermcidin;Survival-promoting peptide;DCD-1	DCD	**-10,3**	**0,00726378**	1,25E+07	9,16E+06	0,00E+00	0,00E+00	ENSG00000161634	0,0	0,0	#DIV/0!
O75027	ATP-binding cassette sub-family B member 7, mitochondrial	ABCB7	**-8,5**	**0,0002364**	8,73E+06	9,20E+06	0,00E+00	0,00E+00	ENSG00000131269	988,7	1016,7	1,03
O95819	Mitogen-activated protein kinase kinase kinase kinase 4	MAP4K4	**-7,2**	**0,00364134**	6,79E+06	8,24E+06	0,00E+00	0,00E+00	ENSG00000071054	6491,1	6429,5	0,99
Q9H330	Transmembrane protein 245	TMEM245	**-2,9**	**0,01595301**	3,38E+08	2,70E+08	1,10E+08	9,69E+07	ENSG00000106771	3621,8	3772,6	1,04
Q5TEJ8	Protein THEMIS2	THEMIS2	**2,0**	**0,03388873**	2,11E+07	2,22E+07	3,74E+07	4,72E+07	ENSG00000130775	2104,8	2254,2	1,07
Q99704	Docking protein 1	DOK1	**2,1**	**0,03791746**	4,91E+07	5,93E+07	9,95E+07	1,23E+08	ENSG00000115325	1512,3	1591,6	1,05
Q8NFF5	FAD synthase;Molybdenum cofactor biosynthesis protein-like region;FAD synthase region	FLAD1	**2,7**	**0,0135542**	3,17E+07	2,96E+07	9,17E+07	7,47E+07	ENSG00000160688	1338,1	1319,8	0,99
O00635	E3 ubiquitin-protein ligase TRIM38	TRIM38	**7,2**	**0,02251141**	0,00E+00	0,00E+00	9,39E+06	5,75E+06	ENSG00000112343	2762,5	2877,4	1,04
P52747	Zinc finger protein 143	ZNF143	**10,4**	**0,02019008**	0,00E+00	0,00E+00	1,39E+07	8,20E+06	ENSG00000166478	850,7	831,5	0,98
Q9H9B1	Histone-lysine N-methyltransferase EHMT1	EHMT1	**14,8**	**0,01199067**	0,00E+00	0,00E+00	1,90E+07	1,22E+07	ENSG00000181090	4675,6	4874,1	1,04
Q5F1R6	DnaJ homolog subfamily C member 21	DNAJC21	**16,1**	**0,00142791**	0,00E+00	0,00E+00	1,56E+07	1,82E+07	ENSG00000168724	2201,5	2248,0	1,02
Q15154	Pericentriolar material 1 protein	PCM1	**16,4**	**0,00305939**	0,00E+00	0,00E+00	1,92E+07	1,53E+07	ENSG00000078674	6562,9	6051,5	0,92
Q8N201	Integrator complex subunit 1	INTS1	**19,8**	**0,00880531**	0,00E+00	0,00E+00	2,50E+07	1,67E+07	ENSG00000164880	8018,0	8024,7	1,00
Q32P28	Prolyl 3-hydroxylase 1	LEPRE1	**21,6**	**0,00836349**	0,00E+00	0,00E+00	2,73E+07	1,83E+07	ENSG00000117385	1782,4	1757,9	0,99
Q8IZ73	RNA pseudouridylate synthase domain-containing protein 2	RPUSD2	**21,8**	**0,03896478**	0,00E+00	0,00E+00	1,36E+07	3,34E+07	ENSG00000166133	681,8	693,3	1,02
O75179	Ankyrin repeat domain-containing protein 17	ANKRD17	**23,4**	**0,00807712**	0,00E+00	0,00E+00	2,95E+07	1,98E+07	ENSG00000132466	6710,8	6373,1	0,95
Q9NX58	Cell growth-regulating nucleolar protein	LYAR	**23,9**	**0,04150994**	0,00E+00	0,00E+00	3,71E+07	1,44E+07	ENSG00000145220	2168,6	2182,5	1,01
Q99856	AT-rich interactive domain-containing protein 3A	ARID3A	**24,6**	**0,00579601**	0,00E+00	0,00E+00	3,02E+07	2,15E+07	ENSG00000116017	2365,6	2441,3	1,03
P16989	Y-box-binding protein 3	YBX3	**25,8**	**0,02238906**	0,00E+00	0,00E+00	3,66E+07	1,84E+07	ENSG00000060138	2727,8	3604,6	1,32
Q96GD4	Aurora kinase B	AURKB	**28,1**	**0,00151378**	0,00E+00	0,00E+00	2,69E+07	3,21E+07	ENSG00000178999	4659,9	4701,1	1,01
Q99700	Ataxin-2	ATXN2	**30,3**	**0,00054331**	0,00E+00	0,00E+00	3,01E+07	3,35E+07	ENSG00000204842	2155,0	2203,9	1,02
O15084	Serine/threonine-protein phosphatase 6 regulatory ankyrin repeat subunit A	ANKRD28	**30,4**	**0,02012491**	0,00E+00	0,00E+00	2,19E+07	4,27E+07	ENSG00000206560	2394,9	2380,2	0,99
Q8TDB6	E3 ubiquitin-protein ligase DTX3L	DTX3L	**30,8**	**0,00108547**	0,00E+00	0,00E+00	2,99E+07	3,48E+07	ENSG00000163840	5050,5	5177,2	1,03
Q07065	Cytoskeleton-associated protein 4	CKAP4	**32,1**	**0,01388882**	0,00E+00	0,00E+00	4,32E+07	2,48E+07	ENSG00000136026	435,7	381,7	0,88
Q9NZ52	ADP-ribosylation factor-binding protein GGA3	GGA3	**34,8**	**0,00235868**	0,00E+00	0,00E+00	3,24E+07	4,07E+07	ENSG00000125447	4476,6	4582,7	1,02
Q9Y5Y4	Prostaglandin D2 receptor 2	PTGDR2	**43,5**	**0,02288745**	0,00E+00	0,00E+00	6,32E+07	2,98E+07	ENSG00000183134	1263,5	1667,1	1,32
P20248	Cyclin-A2	CCNA2	**44,8**	**0,01001673**	0,00E+00	0,00E+00	5,88E+07	3,60E+07	ENSG00000145386	5900,1	5971,3	1,01
Q9NXC5	WD repeat-containing protein mio	MIOS	**58,7**	**0,01285834**	0,00E+00	0,00E+00	7,96E+07	4,48E+07	ENSG00000164654	1041,9	1091,7	1,05
Q96KQ7	Histone-lysine N-methyltransferase EHMT2	EHMT2	**62,2**	**0,00030407**	0,00E+00	0,00E+00	6,81E+07	6,24E+07	ENSG00000204371	4484,3	4449,8	0,99
Q8NE62	Choline dehydrogenase, mitochondrial	CHDH	**65,2**	**0,00134269**	0,00E+00	0,00E+00	7,47E+07	6,21E+07	ENSG00000016391	988,3	896,0	0,91

Supplementary Table 5. List of key antibodies used in the present study.

Antibodies	Supplier	Catalog number and RRID	Dilution
Brilliant Violet 510™ anti-human TCR α/β Antibody	Biolegend	Cat# 306734, RRID:AB_2650821	dilution 1:40
cFluor™ YG584 Anti-Human CD4 (SK3)	Cytek	Cat# R7-20041, RRID:AB_2885083	dilution 1:80
CD25 Monoclonal Antibody (CD25-3G10), PE-Alexa Fluor 700	Thermofisher Scientific	Cat# MHCD2524, RRID:AB_2539740	dilution 1:20
PE/Dazzle™ 594 anti-human CD152 (CTLA-4) Antibody	Biolegend	Cat# 369616, RRID:AB_2632878	dilution 1:20
BUV805 Mouse Anti-Human CD8	BD Biosciences	Cat# 612889, RRID:AB_2833078	dilution 1:80
PerCP/Cyanine5.5 anti-human CD3 Antibody	Biolegend	Cat# 317336, RRID:AB_2561628	1.5ug/mL
Brilliant Violet 510™ anti-human CD4	Sony	Cat# 2187220, RRID:AB_2905654	2.5 μg/ml
PE-Cy™7 Mouse Anti-Human CD25	BD Biosciences	Cat# 561405, RRID:AB_10646034	1.5ug/mL
PE/Cy5 anti-human CD45RA	Sony	Cat# 2120550, RRID:AB_2905655	dilution 1:40
FITC anti-human CD127 (IL-7Rα)	Sony	Cat# 2356560, RRID:AB_2905656	3ug/mL
PE Mouse Anti-Human CTLA-4	BD Biosciences	Cat# 555853, RRID:AB_396176	1.25ug/mL
FOXP3 Monoclonal Antibody (PCH101), APC, eBioscience™	Thermofisher Scientific	Cat# 17-4776-42, RRID:AB_1603280	2.5ug/mL
HELIOS Monoclonal Antibody (22F6), eFluor 450, eBioscience™	Thermofisher Scientific	Cat# 48-9883-42, RRID:AB_2574136	2.5ug/mL
Anti-Human CD134/OX40 (ACT35) - 142Nd - 100 Tests	Fluidigm	Cat# 3142018B, RRID:AB_2905646	2uL per sample
Anti-Human CD278/ICOS (C398.4A) - 175Lu - 100 Tests	Fluidigm	Cat# 3175039B, RRID:AB_2905647	2uL per sample
Anti-Human CD357 (621)-159Tb—100 Tests	Fluidigm	Cat# 3159020B, RRID:AB_2858232	2uL per sample
Anti-Human CD279/PD-1 (EH12.2H7) - 165Ho - 100 Tests	Fluidigm	Cat# 3165042B, RRID:AB_2905648	2uL per sample
Anti-Human TIGIT (MBSA43) - 209Bi - 100 Tests	Fluidigm	Cat# 3209013B, RRID:AB_2905649	2uL per sample
Anti-Human CD366/Tim-3 (F38-2E2) - 169Tm - 100 Tests	Fluidigm	Cat# 3169028B, RRID:AB_2905650	2uL per sample
PE/Dazzle™ 594 anti-human TCR α/β Antibody	Biolegend	Cat# 306726, RRID:AB_2566599	1:50e

Brilliant Violet 421™ anti-human CD152 (CTLA-4) Antibody	Biolegend	Cat# 369606, RRID:AB_2616795	1:20e
PE/Cyanine7 anti-human CD25 Antibody	Biolegend	Cat# 356108, RRID:AB_2561975	1:50e
Brilliant Violet 605™ anti-human CD4 Antibody	Biolegend	Cat# 317438, RRID:AB_11218995	1:50e
Brilliant Violet 711™ anti-human CD8a Antibody	Biolegend	Cat# 310144, RRID:AB_2562906	1:50e
APC-R700 Mouse Anti-Human CD127	BD Biosciences	Cat #565185, RRID:AB_2739099	1:50e
BUV395 Mouse Anti-Human CD45RA	BD Biosciences	Cat# 740315, RRID:AB_2740052	1:50e
HELIOS Monoclonal Antibody (22F6), PerCP-eFluor™ 710	Thermofisher Scientific	Cat# 46-9883-42, RRID:AB_2573924	1:20e
Recombinant Anti-CTLA4 antibody [CAL49]	Abcam	Cat# Ab237712, RRID:AB_2905652	1:1000e
Anti-LRBA antibody produced in rabbit	Sigma	Cat# HPA-023597, RRID:AB_1853256	1:1000e
Anticorps CTLA-4 (F-8)	SantaCruz Biotechnology	Cat# sc-376016, RRID:AB_10988256	1:200e
Mouse monoclonal [SB62a] Anti-Rabbit IgG light chain (HRP)	Abcam	Cat# ab99697, RRID:AB_10673897	1:1000e
Donkey Anti-Mouse IgG Antibody, HRP conjugate, Species Adsorbed	Sigma Aldrich	Cat# AP192P, RRID:AB_11213904	1:1000e
Recombinant Anti-NBEAL2 antibody [EPR14501(B)] - N-terminal	Abcam	Cat# ab187162, RRID:AB_2905645	1:1000e

Supplementary Table 6. List of key reagents used in the present study.

Key reagents	Supplier	Catalog number
Dynabeads™ Human T-Expander CD3/CD28	Thermofisher Scientific	Cat#11141D
Live/Dead Fixable Blue Viability Kit	Thermofisher Scientific	Cat# L23105
Zombie NIR™ Fixable Viability Kit	Biolegend	Cat# 423106
TruStain FcX	Biolegend	Cat# 422302
Sodium Heparin Salt	Sigma Aldrich	Cat# H3149-10KU
Cal-LyseTM Lysing Solution (with formaldehyde and EGTA)	Thermofisher Scientific	Cat# GAS-010S100
PierceTM 16% Formaldehyde (w/v), Methanol-free	Thermofisher Scientific	Cat# 28906
RIPA Lysis and Extraction Buffer	Thermofisher Scientific	Cat# 89900
Phosphatase Inhibitor Cocktail 2	Sigma Aldrich	Cat# P5726
Phosphatase Inhibitor Cocktail 3	Sigma Aldrich	Cat# P0044
Halt™ Protease Inhibitor Cocktail (100X)	Thermofisher Scientific	Cat# 87786
Alt-R® S.p. Cas9 Nuclease V3, 5 mg	Integrated DNA Technologies	Cat# 10000735
Alt-R® Cas9 Electroporation Enhancer, 10 nmol	Integrated DNA Technologies	Cat# 1075916
Nuclease-free Duplex Buffer	Integrated DNA Technologies	Cat# 1072570
DSP (dithiobis(succinimidyl propionate)), Lomant's Reagent	Thermofisher Scientific	Cat# 22586
UltraPure™ 1 M Tris-HCI Buffer, pH 7.5	Thermofisher Scientific	Cat# 15567-027
Sodium chloride solution	Sigma Aldrich	Cat# S5150-1L
n-Octyl-β-D-glucopyranoside, ULTROL® Grade	Sigma Aldrich	Cat# 494460-5gm
Sodium fluoride	Sigma Aldrich	Cat# S7920-100g
Sodium pyrophosphate tetrabasic decahydrate	Sigma Aldrich	Cat# S6422-100g
UltraCruz® Protease Inhibitor Cocktail Tablet	Santa Cruz Biotechnology	Cat# sc-29130
Sodium Orthovanadate (Vanadate)	New England Biolabs	Cat# P0758L
Benzonase® Nuclease	Sigma Aldrich	Cat# E1014-25KU
SepMate kit	Stemcell Technologies	Cat# 85450
EasySep™ Human T Cell Isolation Kit	Stemcell Technologies	Cat# 17951
Foxp3 / Transcription Factor Staining Buffer kit	eBiosciences	Cat# 00-5523-00
Cytofix CytoPerm kit	BD Biosciences	Cat# 554714
Pierce™ BCA Protein Assay Kit	Thermofisher Scientific	Cat# 23225
Human Fas Ligand/TNFSF6 Quantikine ELISA Kit	R&D Systems	Cat# DFL100
Elecsys® Active B12 (holoTC) kit	Roche Diagnostics	Cat# 07 713 207 190
Maxpar Direct Immune Profiling Assay	Fluidigm	Cat# 201325
Maxpar® X8 Antibody Labeling Kit, 162Dy—4 Rxn	Fluidigm	Cat# 201162A
Maxpar® MCP9 Antibody Labeling Kit, 116Cd—4 Rxn	Fluidigm	Cat# 201116A

P2 Primary Cell 4D-NucleofectorTM X Kit S	Lonza	Cat# V4XP-2032
Pierce™ Direct IP Kit	Thermofisher Scientific	Cat# 26148
Chromium Single Cell 3′ Library & Gel Bead Kit v2	10X Genomics	
Chromium Single Cell 3′ Library & Gel Bead Kit v3	10X Genomics	Cat# PN-1000075
Chromium Single Cell 3′ Library & Gel Bead Kit v3.1	10X Genomics	
Pierce™ Spin Cups - Paper Filter	Thermofischer Scientific	Cat# 69700

Supplementary Table 7. List of softwares used in this study.

**Softwares**	**Supplier**	**Link**
Ingenuity Pathway Analysis v57662101	QIAGEN	https://digitalinsights.qiagen.com/products-overview/discovery-insights-portfolio/analysis-and-visualization/qiagen-ipa/
Seurat v3.1	(Stuart et al., 2019)	https://satijalab.org/seurat/
Flowjo v10.7	BD Biosciences	https://www.flowjo.com/solutions/flowjo/downloads
Enrich R	(Chen et al., 2013) (Kuleshov et al., 2016)	https://maayanlab.cloud/Enrichr/
CyTOF software version 6.7.1014	Fluidigm	https://www.fluidigm.com/software
MaxQuant software Version. 1.6.17.0		https://www.maxquant.org/download_asset/maxquant/latest
GraphPAD Prism v9	GraphPad	https://www.graphpad.com/scientific-software/prism/
STRING v.11.5	(Szklarczyk et al., 2020)	https://version-11-5.string-db.org/cgi/network?networkId=bjY9nFy8le23
CellRanger V3.1	10x Genomics	https://support.10xgenomics.com/single-cell-gene-expression/software/downloads/latest

Supplementary Table 8. List of the single guide RNA used for CRISPR-Cas9 knock-down in the present study.

**Guide name**	**Guide sequence (without the PAM)**	**GC %**	**Sens**	**Supplier**	**Format**
LRBA ex3	TTACGTGCCAAGCAGAAGTC	50%	Forward	Integrated DNA Technologies	Custom order in tube, Alt R Cas9 gRNA, 10 nmol
LRBA ex22	CCCACTCATATTTGACTGCA	45%	Forward	Integrated DNA Technologies	Custom order in tube, Alt R Cas9 gRNA, 10 nmol
LRBA ex44 (inefficient)	GACAAATCTCTGAAGTTGGT	40%	Forward	Integrated DNA Technologies	Custom order in tube, Alt R Cas9 gRNA, 10 nmol
LRBA ex50	CAACTCCCCTGTTACTCACG	55%	Reverse	Integrated DNA Technologies	Custom order in tube, Alt R Cas9 gRNA, 10 nmol
LRBA ex55	GTTATCATCTGTTTCCATCG	40%	Forward	Integrated DNA Technologies	Custom order in tube, Alt R Cas9 gRNA, 10 nmol
NBEAL2 ex4	GGCCTGCCTCTATGTTCTCC	60%	Reverse	Integrated DNA Technologies	Custom order in tube, Alt R Cas9 gRNA, 10 nmol
NBEAL2 ex14	TCTTTACCAGCAGCGGCTCA	55%	Forward	Integrated DNA Technologies	Custom order in tube, Alt R Cas9 gRNA, 10 nmol
NBEAL2 ex33	CCTGGAGGGGCTACGCTACA	65%	Forward	Integrated DNA Technologies	Custom order in tube, Alt R Cas9 gRNA, 10 nmol
NBEAL2 ex37	TACGGAACCAGGTGTACTCG	55%	Forward	Integrated DNA Technologies	Custom order in tube, Alt R Cas9 gRNA, 10 nmol
NBEAL2 ex 41	CACCGAGTGGAACTGCCGGT	65%	Reverse	Integrated DNA Technologies	Custom order in tube, Alt R Cas9 gRNA, 10 nmol
NBEAL2 ex 48	GGAAAGCTGCTATTCAGCGG	55%	Forward	Integrated DNA Technologies	Custom order in tube, Alt R Cas9 gRNA, 10 nmol

Supplementary Table 9. List of culture media used in the present study.

Medium name	Recipe
**Complete panserin:**	Panserin 401 medium (PAN BIOTECH, # P04-710401), supplemented with 5% human AB serum, 1% pennicilin/streptomycin (Thermofisher, 15140122) and 1% L-glutamine (Thermofisher, 25030081) and filtered with a unit Nalgene Rapid-Flow single-use sterile filtration unit with PES membrane (Thermofisher, # 568-0010).
**Complete Immunocult (IC-C)**	ImmunoCult - XF T Cell Expansion Medium (StemCell, #10981), supplemented with 2% by volume fetal bovine serum (LifeTechnologies, #10500) and 1% Penicillin/Streptomycin (Pen/Strep) (LifeTechnologies, #15140-122).
**Complete X-Vivo15:**	X-VIVO 15 medium without Gentamycin or Phenol Red (Lonza, #BE02-061Q), supplemented with 1% Penicillin/Streptomycin (LifeTechnologies, #15140-122).

Supplementary Table 10. Recipe for the octyl buffer used in the immunoprecipitation experiments in the present study.

For 100mL solution			
	**Reagents**	**Supplier**	**Reference**
2,5 mL (1M)	25mM Tris	ThermoFischer Scientific	15567-027
3 mL (5M)	150mM NaCl	Sigma Aldrich	S5150-1L
1,2g (1,2%)	n-Octyl-#-D-glucopyranoside	Sigma Aldrich	494460-5gm
64mg	NaF	Sigma Aldrich	S7920-100g
440mg	Sodium pyrophosphate tetrabasic decahydrate	Sigma Aldrich	S6422-100g
2 pastilles	complete protease inhibitor cocktail	ChemCruz	sc-29130
1mL	Na3VO4 100mM	New England Biolabs	P0758L
92mL	H2O		

Supplementary methods

For detailed references of antibodies, key reagents and softwares used in this study, please refer to supplemental Tables S5, S6 and S7.

Cells and plasma isolation

Peripheral blood samples were collected on lithium heparin tubes. Plasma were isolated after 5 min of centrifugation at 1030g, and frozen at -20°C prior to FAS ligand and vitamin B12 assays (see below). Peripheral blood mononuclear cells (PBMC) were isolated by density gradient centrifugation (940g without break for 30 minutes) using Ficoll (Eurobio Scientific). After centrifugation, cells were washed with Phosphate-buffered saline (PBS) (Thermo Fisher scientific). The pellet was resuspended in PBS and cells were centrifuged at 330g for 5 minutes. Finally, the PBMCs pellet was frozen in a freezing medium containing 10% of dimethyl sulfoxide (DMSO) (Sigma Aldrich) and 90% of Fetal Bovine Serum (FBS) (GIBCO, Thermo Fisher scientific). Samples were stored in liquid nitrogen.

T cell isolation

For the isolation of a large quantity of T cells, apheresis rings were obtained from Etablissement Français du Sang (EFS). PBMC were recovered using the SepMate kit (Stemcell Technologies, # 85450), according to the supplier's protocol. T cells were then isolated using the EasySep™ Human T Cell Isolation Kit (Stemcell Technologies, #17951) following the supplier's protocol. The cells were then frozen and cryopreserved at -150°C.

Culture media

The media used in this study are detailed in Supplemental Table S9.

T lymphocytes activation and culture

T cells activation was carried out from the PBMC of patients or of healthy donors coming from the EFS. T cells were activated by binding CD3/CD28 receptors with Dynabeads (Thermofisher, #11141D). Cells were cultured at 1.106/mL/10 µL of Dynabeads in complete Panserin medium. After 3 days of culture, cells were centrifuged on Ficoll and then recultured in complete Panserin with interleukin-2 (IL-2) at 100 international units/mL (U/mL). Every two days, cell expansion was continued by adding Panserin complete with IL-2.

Protein extraction

Proteins were extracted in RIPA lysis buffer (Thermo Fisher, # 89900) supplemented with cocktails of anti-phosphatases (Sigma-Aldrich, #P5726 and #P0044) and anti-protease (Thermo Fisher, # 87786). After 30 min of incubation on ice and centrifugation for 5 min at 3000g, the supernatant containing the extracted proteins was recovered. Proteins were stored at -20°C (for short term use) or -80°C. The concentrations of the protein lysates were measured just before use using the Pierce BCA Protein Assay kit (Thermo Fisher, #23225).

NBEAL2 immunoblotting

Samples were denatured and reduced with NuPAGE LDS Sample buffer 4X (Thermo Fisher, #NP0007) and NuPAGE sample reducing agent 10X (Thermo Fisher, NP009). The migration was carried out on NuPAGE 3-8% Tris Acetate gels (Thermo Fisher, #EA0378BOX) at 150V for 2h. The transfer was done on PVDF membranes (Thermo Fisher, #IB24002) with the iBlot 2 dry transfer system (Thermo Fisher, #IB21001) using a 10min program at 25V constant. The membranes were blocked with 5% milk in TBS (Tris Buffer saline) Tween 0.1% for 1 hour at RT (room temperature) with shaking, then incubated with the recombinant rabbit primary antibody Anti-NBEAL2 [EPR14501(B)] - N-terminal (Abcam, #ab187162) diluted to 1:1000 with stirring at 4° C overnight. After 3 washes with TBS Tween 0.1% (TBST), the anti-rabbit secondary antibody coupled to HRP (Horse Radish Peroxidase) was incubated for 1 hour at RT with stirring at a dilution of 1:10,000th in 5% milk TBST. After 3 washes with TBST, the membrane was incubated for 5 min, at room temperature (RT), in the dark, with the HRP substrate, contained in the commercial solution SuperSignal West Pico PLUS Chemiluminescent Substrate (Thermo Fisher, # 34580), then developed on Amersham hyperfilms (GE Healthcare, #28-9068-37) with CuriX 60 developer (Agfa).

Mass cytometry staining

<u>Antibodies coupling:</u> Anti-CTLA-4 (Cat# 369602, Biolegend) and anti-FAS (#555670, BD Biosciences) antibodies were coupled to 162Dy and 116Cd respectively using the kits #201162A (Fluidigm) and # 201116A (Fluidigm) according to manufacturer protocol.

<u>Samples staining:</u> Immune phenotyping on whole blood was carried out using the Maxpar Direct Immune Profiling kit (Fluidigm, Cat# 201325) with an antibody panel of 30 markers for CyTOF (Cytometry by Time Of Flight) analysis. To these 30 markers, 8 additional antibodies were added to detect FAS and certain immune checkpoints (TIM3, TIGIT, ICOS, GITR, PD-1, CTLA-4). 300 µL of heparinized whole blood were used per labeling. The cells were incubated for 20 min at room temperature (RT) with 3µL of heparin (Sigma Aldrich, Cat# H3149-10KU) at 10,000 U/mL and 5µL of Human TruStain FcX (Biolegend, Cat# 422302), then incubated for 30 min at RT with the antibody cocktail for extracellular labeling (except CTLA-4 for which intracellular labeling was necessary). Blood lysis was perfomed using Cell Cal-lyse buffer (Thermofischer, GAS-010S100) according to manufacturer instructions. After fixation and permeabilization using the Cytofix CytoPerm kit (#554714, BD) the CTLA-4 antibody was incubated with the cells for 30 min at RT. After washing, cells were fixed for 15 min with a 1.6% solution of FA (Formaldehyde, Thermo Fisher, Cat# 28906). Finally, cells were incubated in the Fix&Perm buffer (Fluidigm, Cat# 201325) with the Iridium intercalator at 1:1000 dilution (Fluidigm, Cat# 201325) overnight at 4°C. Cell solutions were frozen at -80°C prior to acquisition.

Acquisition: As previously described, acquisition were performed on Helios mass cytometer *(37)*. For acquisition, cells were washed and resuspended at a concentration of 1.106/mL in Maxpar Cell Acquisition Solution, a high ion concentration solution, and mixed with 10% EQ beads (allowing for calibration automatic device) immediately before the acquisition. The acquisition of the events was carried out on the Helios mass cytometer (Fluidigm) coupled with the CyTOF software version 6.7.1014 (Fluidigm) at the Pitié-792 Salpetrière Cytometry Platform (CyPS). The acquired data were normalised using the Fluidigm normalisation algorithm. Cells were selected by cell selection (Ir191+Ir193+), cell doublets were removed (Time/offset, Time/width, Time/Centre and Time/residual) and dead cells were removed (Ir193+Rh103+). This selection is done automatically with the Pathsetter software.

Data analysis: FCS files containing viable singlet cells were uploaded in R version 4.0.3 using the flowCore package. All files were concatenated in a SingleCellExperiment (SCE) with the SingleCellExperiment package. After quality control (number of cells per sample, expression pattern of all markers across samples), all cells were submitted to clustering using the cluster function (FlowSOM and ConsensusMetaClustering) following recommendation from the CATALYST packages *(38)*. For clustering, k paramater was set to 60 to detect small immune populations. Clusters were then identified based on their expression of type markers according to previous knowledge of immune cell phenotype and following manufacturer instruction from Fluidigm Maxpar Immune Profiling *(38)* (See heatmap in Fig. S2A). Clusters expressing the same type markers were merged into one single immune cell population. Proportion of clusters among all intact viable cells were then defined using appropriate function from CATALYST and compared between groups using Mann-Whitney test in R. To go further in the detail of memory CD4 T cells they were submitted to subclustering using CXCR3, CXCR5, CCR6 and CCR4 as type markers defining Th1-like, Th2-like, Th17-like and Tfh-like cells. Median expression intensity of markers of interest was assessed using the plotPbExprs from CATALYST package. Dimension reduction in UMAPs was performed using runDR function from CATALYST with neighbors set to 15 and minimum distance to 0.4. Marker intensity visualization on UMAPs was performed upon data normalization between 0 and 1. All other visualization was performed using in-house and ggplot2 functions.

Fas Ligand and Vitamin B12 assays

Fas ligand (FASL) and Vitamin B12 dosages were performed on plasma samples. The FasL assay was performed with the ELISA kit (R&D, #DFL00), according to the supplier's recommendations. The vitamin B12 assay was performed using an “ECLIA” electrochemiluminescence immunoassay contained in the Elecsys® Active B12 (holoTC) kit (Roche, # 07 713 207 190), according to the supplier's recommendations.

CRISPR Cas9 experiments

Guide design: using the online tool CRISPOR (website: http://crispor.tefor.net/) (Concordet and Haeussler, 2018), six and five guides were designed for NBEAL2 and LRBA genes respectively and ordered via Integrated DNA Technologies with the format custom Alt R Cas9 gRNA, 10 nmol. The guides are listed in supplemental table S8.

T cells stimulation: T cells previously isolated from blood of healthy EFS donors were thawed and cultured at 500 000 cells/mL in Complete immunocult medium in flask previously coated with anti-CD3 antibody (UCHT1 clone, ThermoFischer Scientific Cat# 16-0038-85) at 0.3 µg/mL. Anti-CD28 antibody (clone 28.2, Biolegend Cat# 302934) was added to the final concentration of 0.06 µg/mL. Cells were incubated at 37°C for 4 days.

Electroporation: On the 4th day, the cells were electroporated. Prior to electroporation, the ribonucleoprotein (RNP) complexes between the Cas9 enzyme (Alt-R Cas9 Nuclease V3, IDT, 10 mg/ml, Mw: 162 kDa, #10000735) and the RNA guides were incubated for 20 min at room temperature, in a buffer containing no nucleases (IDT, #1072570). Electroporation enhancer buffer (IDT, #1075916) was resuspended in 100 nM nuclease-free buffer (IDT, #1072570). Nucleofector supplement was added to Nucleofector solution of P2 Primary Cell 4D X Kit S (LONZA #V4XP-2032). The pre-activated T cells were washed with PBS before electroporation. The cells were then resuspended in P2-4D Nucleofactor™ X Solution: 20µL/1.106 cells/electroporation. 1µL of electroporation enhancer and 5 µL of RNP solution were added for every 20 µL/electroporation. The cells were electroporated using the Amaxa™ 4D-Nucleofector nucleofaction system and the P2-EH100 program. Immediately after electroporation IC-C+IL-2 medium was added to the cells. Cells were incubated for four days at 37°C in IC-C+IL-2 medium.

Culture and cells reactivation: on day 7 following T cell activation, the cells were cultured by diluting them to 1/3 in IC-C+IL-2 medium. On day 8, the cells were restimulated with Dynabeads Human T-Activator CD3/CD28 at 1 beads/16 cells. The cells were transfered in 96-well plates at 100,000 cells/100 µL of X-Vivo15 complete medium (Lonza, #BE02-061Q) and incubated at 37°C.

Cytometry staining and protein extraction: each day post re-stimulation, cells were labeled to analyze the expression of the immune checkpoints using the Cytek® Aurora cytometer CS according to the protocol described in cytometry staining paragraph. In parallel, after 72 hours after reactivation, cells were lysed for protein extraction as described in the "protein extraction" method paragraph.

Flow cytometry staining

Treg staining: PBMC were thawed, washed with PBS and stained. Extracellular staining was performed 30 min on ice using antibodies anti-CD3-PerCP-Cy5.5 ; CD4 BV510 ; CD127-FITC ; CD45RA Pe-Cy5 ; CD25 PE-Cy7. Cells were permeabilized and fixed with the kit Foxp3 / Transcription Factor Staining Buffer (eBioscience™ #00-5523-00), according to manufacturer

instructions. Intracellular staining was performed 1h on ice using antibodies anti-CTLA-4 PE; FoxP3 APC; Helios eF450. Cells were resuspended in PBS and analyzed on the BD LSRFortessa™ X-20 SORP Cell Analyzer cytometer.

Activated T cells after CRISPR knock-down: Activated T cells were stained after electroporation and re-activation. Cells were stained for viability using LIVE/DEAD™ Fixable Blue Dead Cell Stain Kit, for UV excitation (Thermofischer, #L23105). Extracellular staining was carried out 30 min on ice using TCR – BV510, CD4 – YG594, CD8 – BV805, CD25 – PE-AF700. Cells were fixed and permeabilized using the Cytofix CytoPerm kit (#554714, BD). Intracellular staining was performed with antibody anti-CTLA-4 Pe-Dazzle. Cells were analyzed on the Aurora cytometer.

Activated T cells: After CD3/CD28 activation and 12 days of cultures, activated T cells were stained with Zombie NIR, TCRab, CD4, CD25, CD8. Cells were fixed and permeabilized using the Cytofix CytoPerm kit (#554714, BD). Intracellular staining was performed with antibody anti-CTLA-4 BV421. Cells were analyzed on the Sony Biotechnology SP6800 Spectral Analyzer.

The mean of fluorescence intensities (MFI) of each sample was normalized using the mean MFI of the internal control (healthy donors of the day) and the following equation for the test samples (patients and healthy donors) : MFI_{sample} / $MFI_{mean\_controls\_of\_the\_day}$.

CTLA-4 Immunoprecipitation

Cells: T cells isolated from the blood of healthy donors were used. A comparison between non-activated T cells and T cells activated was carried out. Unstimulated T cells express little CTLA-4 and served as a negative control for immunoprecipitation. Cell activation was performed with Dynabeads CD3/CD28 (ThermoFisher, # 11141D): 1 bead for 30 cells. Cell cultures were made at 1.106 cells/mL at 37°C. in complete immunocult medium. After four days of culture, the activated T cells were counted and re-suspended in complete Immunocult medium with IL-2. On the 5th day, the cells were lysed. On the same day, the non-activated T cells were thawed and equilibrated for 1 hour at 37° C.

Antibody coupling: the antibody anti-CTLA-4 (ref Ab251599, Abcam) at 0.998 mg/mL was coupled to agarose beads using the kit Thermo Pierce Direct IP kit (ThermoFisher, Cat# 26148) according to the supplier's instructions. After the last two washes, the beads were resuspended in 400µL of Conditioning Buffer 1x and stored at 4°C for 24 h.

Cross-linking and cell lysis: a reversible cross-linker was used to preserve protein-protein interactions: dithiobis[succinimidylproprionate] (DSP, Thermofisher, Cat# 22586). DSP was diluted just before use in DMSO to a concentration of 10mg/mL. It was then diluted in PBS 1:25 (100 mL of DSP at 1 mM final). Cells were washed in cold PBS and resuspended in the 1mM DSP solution. Cells were incubated with the cross-linker for 30 min, at room temperature (RT), under agitation. Then 750 µL of 1M TRIS were added and stirred for 3 min at RT. After centrifugation (300g, 5min, 4°C) the pellet was washed with cold PBS then the cells were centrifuged (600g, 5min, 4°C). The

pellets were resuspended in Octyl lysis buffer (see Supplemental Table S10). The solutions were sonicated 3 times for 10 seconds. 10 µL of benzonase (Sigma, #E1014-25KU) was added. The lysates were incubated for 2 hours under agitation at 4°C. The lysates were centrifuged at 15000 rcf, at 4°C for 15 min, then the supernatant was transferred to a new tube. A part of the lysate (100 µL, INPUT fraction) was frozen. Protein dosages were performed using the Pierce™ BCA Protein Assay Kit (Thermofisher, #23225).

Immunoprecipitation: the concentrations of the lysates were adjusted if necessary, according to the results of the protein assay. 15 µg of beads coupled to the antibody were added to each condition. The lysates were shaken on a wheel in a cold room overnight. Tubes were centrifuged for 5 min, 1200g, 4°C. 100 µL of the supernatants (Flow-through) were retrieved. The rest of the supernatants were aspirated, and the pellets were washed with 10 mL of cold lysis buffer, then centrifuged at 1200g, 5 min, at 4°C. Pellets were re-suspended in 400 µL of cold lysis buffer and applied to Pierce Spin Cups columns (Thermofisher, #69700). Three washes were carried out with 400 µL of lysis buffer with a 2 min centrifugation at 4°C 1200g. A solution of 1x Blue loading buffer (Cell Signaling Technologies, #56036S diluted in water) was prepared. 50 µL were added to each column retaining the beads and incubated for 20 min at 60° C., under agitation. After centrifugation (5min, 5000g, RT), the eluate was recovered and 3µL of β-mercaptoethanol (Biorad, ref #1610710) was added. The eluates were then reduced for 5 min at 60° C, under agitation, then loaded on the gel.

Immunoblotting: the fractions of the initial protein lysates (input) as well as the fractions of the final eluates (flow-through) were denatured and reduced with Blue loading buffer and β-mercaptoethanol for 20 min at 60° C, under agitation. 4-15% midi gels (Biorad, #5671083) were used and migration of the samples for 1h30 at 180V was carried out in a 1x Tris Glycine SDS buffer (Biorad, #161-0732). Gels were soaked in a 20% ethanol bath for 2 min before transfer. The transfers were performed on nitrocellulose membranes (Trans-Blot Turbo Midi 0.2 µm Nitrocellulose Transfer Packs, Biorad, #1704159) with the 2.5A, 25V, 15 min program on the Trans-Blot device. Blot® Turbo™ Transfer System (Biorad, #1704150). Staining with Ponceau red (Sigma, #P7170-1L) was performed, then this staining was washed with TBS 1X. The membranes were saturated with a 10% solution of milk (PanReac AppliChem, #A0830) in 1X TBST (Cell signaling, #9997). The incubations of the primary antibodies were carried out overnight at 4°C in a 10% milk solution, TBST 1X: anti-NBEAL2 (Abcam, #Ab187162): dilution 1:1000 or CTLA-4 (Abcam, #Ab237712): dilution 1:1000 or CTLA-4 (SantaCruz Biotechnology, #sc-376016): dilution 1:200 or LRBA (Sigma Prestige, #HPA-023597): dilution 1 :1000. After two washes with 1X TBST and two washes with 1X TBS, the secondary antibodies coupled to HRP were diluted in the 10% milk solution and incubated for 1 hour, at RT, under agitation: Mouse monoclonal [SB62a] Anti-Rabbit IgG light chain (HRP), (Abcam, #ab99697): dilution 1:1000 or Goat anti-mouse IgG1 (HRP) (Sigma Aldrich, #AP192P): dilution 1:1000. After incubation, washes were performed. The substrates SuperSignal West Dura (Thermo #859024 + 859025) or Femto (Thermo #1859022 + 1859023) were used. Membranes were revealed on ECL hyperfilms (Amersham, #28906836) on

the Optimax X-ray film processor (Protec) or using a scanning system Chemidoc MP imaging (Biorad). When a second revelation was necessary, membranes were stripped with a stripping buffer (Millipore, #2060-1) diluted in water to the 10th, incubation 15 min with agitation, at RT. Four washes were carried out then the membranes were blocked again with a 10% milk solution for 1 hour with stirring at RT allowing a new incubation with a primary antibody can then be carried out.

NBEAL2 immunoprecipitation

For NBEAL2 immunoprecipitation experiments, PBMC from healthy donor and NBEAL2 patients were activated for 7 days with CD3/CD28 beads in Panserin complete medium (as described above). The same protocol of immunoprecipitation was used. The agarose beads were coupled to the anti-NBEAL2 rabbit (Abcam, Cat# ab250919) using the Thermo Pierce Direct IP kit (ThermoFisher, ref 26148) according to supplier's instructions.

Proteomics analysis after NBEAL2 immunoprecipitation

In gel digestion: NBEAL2 immunoprecipitation fractions were separated by SDS-PAGE (4-20%) under reducing conditions. Six gel bands covering the entire gel area were excised, reduced with DTT, alkylated with iodoacetamide and in-gel digested overnight with trypsin. Peptides were extracted with 50 mM ammonium bicarbonate and 50 % acetonitrile in 0.2 % formic acid, dried by evaporation in a speed-vac concentrator and resuspended in 60 µl of 0.2% formic acid for injection in LC-MS/MS.

Liquid Chromatography Mass Spectrometry analysis (LC-MS/MS): LC-MS/MS analyses were performed using a nano-ACQUITY Ultra-Performance LC system (Waters, Milford, MA) coupled to an Orbitrap Fusion Tribrid mass spectrometer (Thermo Fisher Scientific, San Jose, CA). LC separation was performed with a trapping column (nano-Acquity Symmetry C18, 100 Å, 5 µm, 180 µm x 20 mm) at 15 µl/min flow rate and an analytical column (nano-Acquity BEH C18, 130 Å, 1.7 µm, 75 µm x 250 mm) directly coupled to the ion source. The mobile phases for LC separation were 0.2% (v/v) formic acid in LC-MS grade water (solvent A) and 0.2% (v/v) formic acid in acetonitrile (solvent B). Peptides were separated at a 300 nl/min constant flow rate with a linear gradient of 5-85% solvent B for 120min for global proteome analysis. A full MS1 survey scan was acquired in the Orbitrap for m/z 325-1200 at a 50 ms maximum filling time and 2e5 ions. Resolution was set to 120k at m/z 200. Fragmentation was performed in HCD fragmentation cell (collision energy at 26%), with isolation of precursor ions in the quadrupole. Target ions previously selected for fragmentation were dynamically excluded for 50s with a relative mass window of ±10 ppm. The MS/MS selection threshold was set to 5e3 ion counts. The detection was performed in the Ion Trap with an Automatic Gain Control (AGC) of 2e4 target value and a 50 ms maximum injection time. Each sample was injected twice (technical replicate).

Data processing: DDA data were processed with MaxQuant software (Ver. 1.6.17.0, Max-Planck Institute of Biochemistry, Department of Proteomics and Signal Transduction, Munich). Database searching was performed against the human FASTA database downloaded from UniProtKB/Swiss-Prot. Interrogation of the databank was based on the following criteria: precursor mass tolerance of 7 ppm; fragment ions mass tolerance of 0.6 Da and 2 maximum missed cleavages with trypsin as the enzyme. Search parameters for post-translational modifications were variable modifications of N-acetylation on protein N-terminal residues, oxidation on methionine residues and pyro-Glu modification on glutamine residues. The matching between runs was also checked out. All the other parameters were MaxQuant default parameters. Protein intensities were exported from MaxQuant proteinGroups file. Missing values were replaced by the minimum value of each acquisition. Medians were calculated over the technical replicates.

Proteins selection: Seventy-six proteins with the highest probability to interact with NBEAL2 were selected as described in supplemental figure S3.

STRING and EnrichR analysis: the 74 selected proteins were mapped using the STRING database *(23) (39)*. Enrichment analysis was performed using EnrichR *(26) (40)* webtool and the Jensen Tissue library *(41)(42)*.

Treg and CD25- CD4+ T cells sorting

Treg and CD25- CD4+ T cells were sorted from fresh healthy donor PBMC, using the EasySep™ Human CD4+CD127lowCD25+ Regulatory T Cell Isolation Kit. Small amount of the sorted cells and initial PBMC fractions were stained as described in the Treg staining paragraph with TCRab – BV510, CD4 BV605, CD8a BV711, CD25 PE/Cy7, CD127 AFR700, CTLA-4 PE, FoxP3 APC, CD45RA BUV395 and Helios PerCPeF710 and acquired on Cytek® Aurora cytometer CS. After sorting, the Treg fraction and part of the CD25- CD4+ were lysed in octyl buffer, as described above. The rest of the CD25- CD4+ T cells were activated with CD3/CD28 beads for 5 days in Immunocult complete medium. After activation, cells were lysed. Immunoblotting was performed using 12-230kDa separation module on Jess system (Biotechne).

Single-cell RNA sequencing

The scRNA-seq libraries were generated using Chromium Single Cell 3′ Library & Gel Bead Kit v.2 or kit v.3 or kit v.3.1 as previously described *(37)*, (10x Genomics) according to the manufacturer's protocol. Briefly, cells were counted, diluted at 1000 cells/µL in PBS+0,04% and 20 000 cells were loaded in the 10x Chromium Controller to generate single-cell gel-beads in emulsion. After reverse transcription, gel-beads in emulsion were disrupted. Barcoded complementary DNA was isolated and amplified by PCR. Following fragmentation, end repair and A-tailing, sample indexes were added during index PCR. The purified libraries were sequenced on a Novaseq (Illumina) with 26

cycles of read 1, 8 cycles of i7 index and 98 cycles of read 2 (for kit v.2.) or with 28 cycles of read 1, 8 cycles of i7 index and 91 cycles of read 2 (for kit v.3 and v.3.1).

Single-cell RNA sequencing analysis

Single-cell RNA sequencing analyses were performed as previously described *(37)*. Sequencing reads were demultiplexed and aligned to the human reference transcriptome (GRCh38 directly download from 10x), using the CellRanger Pipeline (v3.0.2). The unfiltered raw UMI counts from Cellranger were loaded into Seurat v4.0.4 *(43)* for quality control, data integration and downstream analyses. Duplets, empty sequencing beads and apoptotic cells were removed by filtering out cells with fewer than 500 features or a mitochondrial content higher than 20%. Data from each sample were normalized and scaled using the sctransform method, and batch effect between samples was corrected using Seurat's FindIntegratedAnchors. The level of ambient mRNA present in the samples was calculated using soupX with default settings. For each sample, the raw and filtered matrices from cellranger were loaded using the load10X function. The contamination fraction was calculated by combining the results of the automated method (autoEstCont function) with the manual one, in which immunoglobulin genes and other genes linked to contamination (PPBP, HBB and HBA1) were passed to estimateNonExpressingCells to estimate the contamination values. On this integrated dataset, we computed the principal component analysis on the 3000 most variable genes. UMAP was carried out using the 30 most significant PCs, and community detection was performed using the graph-based modularity-optimization Louvain algorithm from Seurat's FindClusters function with a 1.8 resolution. Cell type labels were assigned to resulting clusters based on a manually curated list of marker genes as well as previously defined signatures of the well-known PBMC subtypes *(44)*. All clusters were annotated, and 134,776 cells were kept for further analysis. Differential expression was performed on different groups (all PBMCs, Myeloid cells, T cells, B cells, and their sub-populations), using the FindMarkers function of Seurat on the RNA assay with default parameters (Wilcoxon testing with Bonferroni correction). Only genes with adjusted p-values < 0.05 were selected as significant. The lists of differentially expressed genes were further divided into UP and DOWN regulated genes based on the avg_log2FC; avg_log2FC >0 for the UP regulated genes and avg_log2FC <0 for the DOWN regulated ones. All differentially expressed genes with a avg_log2FC > 1.2 or <-1.2 were selected for pathways analysis with the Ingenuity pathway analysis v57662101 software (IPA, QIAGEN Inc.) *(45)*. Heatmaps were extracted from the comparison module in IPA. Pathways with an absolute z-score lower than 2 or a Bonferroni-Hochberg corrected p values higher than 0.05 were filtered out. From the Molecular Signatures Database (MsigDB), the genes in the IL6/JAK STAT3 gene set (HALLMARK_IL6_JAK_STAT3_SIGNALING) were used to calculate the signature scores using the AddModuleScore function from Seurat, and dot plots were used to group and visualize the change in signature signal between conditions (e.g. cell type, gene). Single-cell RNA-sequencing data are available at the GEO accession number GSE196606.

Statistical analysis

Comparisons among groups were performed using nonparametric Mann-Whitney tests or Kruskall-Wallis tests for multiple comparison, corrected with a Dunn test or Wilcoxon signed-rank tests using the Prism 9 software (GraphPad). Each test is specified in the figure legends. *p*-values are shown when relevant (*) *p*-value < 0.05; (**) *p*-value < 0.01; (***) *p*-value < 0.005 ; (****) *p*-value < 0.0001.
