# Supplementary material for: NBEAL2 deficiency in humans leads to low CTLA-4 expression in activated conventional T cells
Source: Nat Commun. 2023 Jun 22;14:3728. doi: 10.1038/s41467-023-39295-7 (PMC10287742; doi:10.1038/s41467-023-39295-7)
Supplement: Supplementary file 3 — Reporting Summary [file 41467_2023_39295_MOESM3_ESM.pdf]

nature portfolio

Corresponding author(s): Dr. Frédéric Rieux-Laucat

Last updated by author(s): 2023/05/18

Reporting Summary

Nature Portfolio wishes to improve the reproducibility of the work that we publish. This form provides structure for consistency and transparency in reporting. For further information on Nature Portfolio policies, see our Editorial Policies and the Editorial Policy Checklist.

Statistics

For all statistical analyses, confirm that the following items are present in the figure legend, table legend, main text, or Methods section.

n/a	Confirmed	
[ ]	[x]	The exact sample size (*n*) for each experimental group/condition, given as a discrete number and unit of measurement
[ ]	[x]	A statement on whether measurements were taken from distinct samples or whether the same sample was measured repeatedly
[ ]	[x]	The statistical test(s) used AND whether they are one- or two-sided<br>*Only common tests should be described solely by name; describe more complex techniques in the Methods section.*
[x]	[ ]	A description of all covariates tested
[x]	[ ]	A description of any assumptions or corrections, such as tests of normality and adjustment for multiple comparisons
[ ]	[x]	A full description of the statistical parameters including central tendency (e.g. means) or other basic estimates (e.g. regression coefficient) AND variation (e.g. standard deviation) or associated estimates of uncertainty (e.g. confidence intervals)
[ ]	[x]	For null hypothesis testing, the test statistic (e.g. *F*, *t*, *r*) with confidence intervals, effect sizes, degrees of freedom and *P* value noted<br>*Give P values as exact values whenever suitable.*
[x]	[ ]	For Bayesian analysis, information on the choice of priors and Markov chain Monte Carlo settings
[x]	[ ]	For hierarchical and complex designs, identification of the appropriate level for tests and full reporting of outcomes
[x]	[ ]	Estimates of effect sizes (e.g. Cohen's *d*, Pearson's *r*), indicating how they were calculated

Our web collection on statistics for biologists contains articles on many of the points above.

Software and code

Policy information about availability of computer code

Data collection	No data collection was performed.
Data analysis	Mass cytometry data, interactome and single cell RNA Sequencing analyses are described in the supplemental methods and figures. Software and algorithms already described in the literature were used, as detailed in supplemental methods and supplemental Table S7. The following software were used: Ingenuity Pathway Analysis v57662101, Seurat v3.1, Flowjo v10.7, EnrichR online database, CyTOF software version 6.7.1014, MaxQuant software Version. 1.6.17.0, GraphPAD Prism v9, STRING online database v.11.5 and CellRanger V3.1.

For manuscripts utilizing custom algorithms or software that are central to the research but not yet described in published literature, software must be made available to editors and reviewers. We strongly encourage code deposition in a community repository (e.g. GitHub). See the Nature Portfolio guidelines for submitting code & software for further information.

Data

Policy information about availability of data

All manuscripts must include a data availability statement. This statement should provide the following information, where applicable:

- Accession codes, unique identifiers, or web links for publicly available datasets
- A description of any restrictions on data availability
- For clinical datasets or third party data, please ensure that the statement adheres to our policy

The new NBEAL2 variant sequence is deposited in National Center for Biotechnology Information. ClinVar; [VCV002443319.1], https://www.ncbi.nlm.nih.gov/clinvar/variation/VCV002443319.1.

Single-cell RNA-sequencing data are available at the GEO accession number GSE196606, https://www.ncbi.nlm.nih.gov/geo/query/acc.cgi?acc=GSE196606.
The mass spectrometry proteomics data have been deposited to the ProteomeXchange Consortium via the jPOST partner repository with the dataset identifier PXD042180 (https://proteomecentral.proteomexchange.org/cgi/GetDataset?ID=PXD042180) and JPST002157 (https://repository.jpostdb.org/entry/JPST002157), which is publicly available.
Interactome dataset visualized on STRING database is available here: https://version-11-5.string-db.org/cgi/network?networkId=bjY9nFy8le23

Human research participants

Policy information about studies involving human research participants and Sex and Gender in Research.

Reporting on sex and gender	Sex and/or gender based analyses were not performed.
Population characteristics	Population characteristics are described in Figure 1A and supplemental table 1.
Recruitment	Informed consents have been obtained for all patients before the study. Participants were recruited on the basis of their disease (grey platelet syndrome).
Ethics oversight	Before the study, all patients signed informed consents approved by the CERAPH-Centre (IRB: #00011928). The biological samples are part of Inserm UMR1163/Imagine collection declared to the French Ministère de la recherche (CODECOH n° DC-2020-3994).

Note that full information on the approval of the study protocol must also be provided in the manuscript.

Field-specific reporting

Please select the one below that is the best fit for your research. If you are not sure, read the appropriate sections before making your selection.

[x] Life sciences [ ] Behavioural & social sciences [ ] Ecological, evolutionary & environmental sciences

For a reference copy of the document with all sections, see nature.com/documents/nr-reporting-summary-flat.pdf

Life sciences study design

All studies must disclose on these points even when the disclosure is negative.

Sample size	The sample sizes for each group (patients or healthy donors) are specified in the figures legends. No sample size calculation were performed prior to the experiment.
Data exclusions	No data were excluded.
Replication	Immune phenotype as well as single cell RNA sequencing were performed once for each patient, indeed rare disease patients cells are extremely precious material, so we decided to analyse the maximum number of patients cells available for several experiments.
Randomization	Randomization was not relevant to this study because we compared patients presenting with one specific disease to healthy donors.
Blinding	Blinding was not relevant to this study because we compared patients presenting with one specific disease to healthy donors.

Reporting for specific materials, systems and methods

We require information from authors about some types of materials, experimental systems and methods used in many studies. Here, indicate whether each material, system or method listed is relevant to your study. If you are not sure if a list item applies to your research, read the appropriate section before selecting a response.

Materials & experimental systems

n/a	Involved in the study	
[ ]	[x]	Antibodies
[x]	[ ]	Eukaryotic cell lines
[x]	[ ]	Palaeontology and archaeology
[x]	[ ]	Animals and other organisms
[x]	[ ]	Clinical data
[x]	[ ]	Dual use research of concern

Methods

n/a	Involved in the study	
[x]	[ ]	ChIP-seq
[ ]	[x]	Flow cytometry
[x]	[ ]	MRI-based neuroimaging

Antibodies

Antibodies used

Brilliant Violet 510™ anti-human TCR α/β Antibody, Biolegend, Cat# 306734, RRID:AB_2650821, dilution 1:40
cFluor™ YG584 Anti-Human CD4 (SK3), Cytek, Cat# R7-20041, RRID:AB_2885083, dilution 1:80
CD25 Monoclonal Antibody (CD25-3G10), PE-Alexa Fluor 700, Thermofisher Scientific, Cat# MHCD2524, RRID:AB_2539740, dilution 1:20
PE/Dazzle™ 594 anti-human CD152 (CTLA-4) Antibody, Biolegend, Cat# 369616, RRID:AB_2632878, dilution 1:20
BUV805 Mouse Anti-Human CD8, BD Biosciences, Cat# 612889, RRID:AB_2833078, dilution 1:80
PerCP/Cyanine5.5 anti-human CD3 Antibody, Biolegend, Cat# 317336, RRID:AB_2561628, 1.5ug/mL
Brilliant Violet 510™ anti-human CD4, Sony, Cat# 2187220, RRID:AB_2905654, 2.5 µg/ml
PE-Cy™7 Mouse Anti-Human CD25, BD Biosciences, Cat# 561405, RRID:AB_10646034, 1.5ug/mL
PE/Cy5 anti-human CD45RA, Sony, Cat# 2120550, RRID:AB_2905655, dilution 1:40
FITC anti-human CD127 (IL-7Rα), Sony, Cat# 2356560, RRID:AB_2905656, 3ug/mL
PE Mouse Anti-Human CTLA-4, BD Biosciences, Cat# 555853, RRID:AB_396176, 1.25ug/mL
FOXP3 Monoclonal Antibody (PCH101), APC, eBioscience™, Thermofisher Scientific, Cat# 17-4776-42, RRID:AB_1603280, 2.5ug/mL
HELIOS Monoclonal Antibody (22F6), eFluor 450, eBioscience™, Thermofisher Scientific, Cat# 48-9883-42, RRID:AB_2574136, 2.5ug/mL
Anti-Human CD134/OX40 (ACT35) - 142Nd - 100 Tests, Fluidigm, Cat# 3142018B, RRID:AB_2905646, 2uL per sample
Anti-Human CD278/ICOS (C398.4A) - 175Lu - 100 Tests, Fluidigm, Cat# 3175039B, RRID:AB_2905647, 2uL per sample
Anti-Human CD357 (621)-159Tb—100 Tests, Fluidigm, Cat# 3159020B, RRID:AB_2858232, 2uL per sample
Anti-Human CD279/PD-1 (EH12.2H7) - 165Ho - 100 Tests, Fluidigm, Cat# 3165042B, RRID:AB_2905648, 2uL per sample
Anti-Human TIGIT (MBSA43) - 209Bi - 100 Tests, Fluidigm, Cat# 3209013B, RRID:AB_2905649, 2uL per sample
Anti-Human CD366/Tim-3 (F38-2E2) - 169Tm - 100 Tests, Fluidigm, Cat# 3169028B, RRID:AB_2905650, 2uL per sample
PE/Dazzle™ 594 anti-human TCR α/β Antibody, Biolegend, Cat# 306726, RRID:AB_2566599, 1:50e
Brilliant Violet 421™ anti-human CD152 (CTLA-4) Antibody, Biolegend, Cat# 369606, RRID:AB_2616795, 1:20e
PE/Cyanine7 anti-human CD25 Antibody, Biolegend, Cat# 356108, RRID:AB_2561975, 1:50e
Brilliant Violet 605™ anti-human CD4 Antibody, Biolegend, Cat# 317438, RRID:AB_11218995, 1:50e
Brilliant Violet 711™ anti-human CD8a Antibody, Biolegend, Cat# 310144, RRID:AB_2562906, 1:50e
APC-R700 Mouse Anti-Human CD127, BD Biosciences, Cat #565185, RRID:AB_2739099, 1:50e
BUV395 Mouse Anti-Human CD45RA, BD Biosciences, Cat# 740315, RRID:AB_2740052, 1:50e
HELIOS Monoclonal Antibody (22F6), PerCP-eFluor™ 710, Thermofisher Scientific, Cat# 46-9883-42, RRID:AB_2573924, 1:20e
Recombinant Anti-CTLA4 antibody [CAL49], Abcam, Cat# Ab237712, RRID:AB_2905652, 1:1000e
Anti-LRBA antibody produced in rabbit, Sigma, Cat# HPA-023597, RRID:AB_1853256, 1:1000e
Anticorps CTLA-4 (F-8), SantaCruz Biotechnology, Cat# sc-376016, RRID:AB_10988256, 1:200e
Mouse monoclonal [SB62a] Anti-Rabbit IgG light chain (HRP), Abcam, Cat# ab99697, RRID:AB_10673897, 1:1000e
Donkey Anti-Mouse IgG Antibody, HRP conjugate, Species Adsorbed, Sigma Aldrich, Cat# AP192P, RRID:AB_11213904, 1:1000e
Recombinant Anti-NBEAL2 antibody [EPR14501(B)] - N-terminal, Abcam, Cat# ab187162, RRID:AB_2905645, 1:1000e

Validation

Titration of antibodies were performed for flow cytometry antibodies, or the recommended concentration by the supplier was applied. For western blot antibodies, dilutions were validated from suppliers recommendations. Moreover, Abcam used recombinant antibodies and "extensive validation methods, including knock-out validation, to ensure the specificity of our recombinant antibodies."

Flow Cytometry

Plots

Confirm that:

☒ The axis labels state the marker and fluorochrome used (e.g. CD4-FITC).

☒ The axis scales are clearly visible. Include numbers along axes only for bottom left plot of group (a 'group' is an analysis of identical markers).

☒ All plots are contour plots with outliers or pseudocolor plots.

☒ A numerical value for number of cells or percentage (with statistics) is provided.

Methodology

Sample preparation

Sample preparations are described in details the supplemental material and methods.
Treg staining: PBMC were thawed, washed with PBS and stained. Extracellular staining was performed 30 min on ice. Cells were permeabilized and fixed with the kit Foxp3 / Transcription Factor Staining Buffer (eBioscience™ #00-5523-00), according to manufacturer instructions. Intracellular staining was performed 1h on ice. Cells were resuspended in PBS.
Activated T cells after CRISPR knock-down: Activated T cells were stained after electroporation and re-activation. Cells were stained for viability using LIVE/DEAD™ Fixable Blue Dead Cell Stain Kit. Extracellular staining was carried out 30 min on ice. Cells were fixed and permeabilized using the Cytofix CytoPerm kit (#554714, BD). Intracellular staining was performed 1h on ice. Cells were resuspended in FACS buffer.
Activated T cells: After CD3/CD28 activation and 12 days of cultures, activated T cells were stained with Zombie NIR viability, 30 min on ice. Extracellular staining was performed 30 min on ice. Cells were fixed and permeabilized using the Cytofix CytoPerm kit (#554714, BD). Intracellular staining was performed 1h on ice. Cells were resuspended in FACS buffer.

Instrument	Becton Dickinson LSRFortessa X-20 or Sony Biotechnology SP6800 Spectral Analyzer or Cytek Aurora cytometer
Software	FLowJo software was used (version 10).
Cell population abundance	Treg and CD25- CD4+ T cells were sorted from fresh healthy donor PBMC, using the EasySep™ Human CD4+CD127lowCD25+ Regulatory T Cell Isolation Kit. Small amount of the sorted cells and initial PBMC fractions were stained with anti TCRab, CD4, CD25, CD127, CTLA-4 and FoxP3 antibodies. The cell population abundances were superior to 90%.
Gating strategy	Data provided in the main figures and in supplemental material and methods. Tregs were gated on CD3+ / CD4+ / CD25+ CD127low or CD3+ / CD4+ / Helios+ FoxP3+ stainings. Activated T cells were gated on alive TCRab+ cells.

☒ Tick this box to confirm that a figure exemplifying the gating strategy is provided in the Supplementary Information.
